# Insecticidal Triterpenes in Meliaceae: Plant Species, Molecules, and Activities: Part II (*Cipadessa*, *Melia*)

**DOI:** 10.3390/ijms23105329

**Published:** 2022-05-10

**Authors:** Meihong Lin, Xiaoyang Bi, Lijuan Zhou, Jiguang Huang

**Affiliations:** Key Laboratory of Natural Pesticides and Chemical Biology, Ministry of Education, South China Agricultural University, Guangzhou 510642, China; 24628@noposion.com (M.L.); xiaoyangbi@stu.scau.edu.cn (X.B.)

**Keywords:** Meliaceae, triterpenoid molecules, insecticidal activities

## Abstract

Plant-originated triterpenes are important insecticidal molecules. Research on the insecticidal activity of molecules from Meliaceae plants has always been a hotspot due to the molecules from this family showing a variety of insecticidal activities with diverse mechanisms of action. In this paper, we discussed 116 triterpenoid molecules with insecticidal activity from 22 plant species of five genera (*Cipadessa*, *Entandrophragma*, *Guarea*, *Khaya*, and *Melia*) in Meliaceae. In these genera, the insecticidal activities of plants from *Entandrophragma* and *Melia* have attracted substantial research attention in recent years. Specifically, the insecticidal activities of plants from *Melia* have been systemically studied for several decades. In total, the 116 insecticidal chemicals consisted of 34 ring-intact limonoids, 31 ring-seco limonoids, 48 rearranged limonoids, and 3 tetracyclic triterpenes. Furthermore, the 34 ring-intact limonoids included 29 trichilin-class chemicals, 3 azadirone-class chemicals, and 1 cedrelone-class and 1 havanensin-class limonoid. The 31 ring-seco limonoids consisted of 16 C-seco group chemicals, 8 B,D-seco group chemicals, 4 A,B-seco group chemicals, and 3 D-seco group chemicals. Furthermore, among the 48 rearranged limonoids, 46 were 2,30-linkage group chemicals and 2 were 10,11-linkage group chemicals. Specifically, the 46 chemicals belonging to the 2,30-linkage group could be subdivided into 24 mexicanolide-class chemicals and 22 phragmalin-class chemicals. Additionally, the three tetracyclic triterpenes were three protolimonoids. To sum up, 80 chemicals isolated from 19 plant species exhibited antifeedant activity toward 14 insect species; 18 chemicals isolated from 17 plant species exhibited poisonous activity toward 10 insect species; 16 chemicals isolated from 11 plant species possessed growth-regulatory activity toward 8 insect species. In particular, toosendanin was the most effective antifeedant and insect growth-regulatory agent. The antifeedant activity of toosendanin was significant. Owing to its high effect, toosendanin has been commercially applied. Three other molecules, 1,3-dicinnamoyl-11-hydroxymeliacarpin, 1-cinnamoyl-3-methacryl-11-hydroxymeliacarpin, and 1-cinnamoyl-3-acetyl-11-hydroxymeliacarpin, isolated from *Melia* *azedarach*, exhibited a highly poisonous effect on *Spodoptera littoralis*; thus, they deserve further attention.

## 1. Introduction

Currently, chemical insecticides are still undoubtedly the most useful method to control insect pests. However, it is also clear that the residue of certain insecticides could lead to some possible negative impacts on human health, food safety, and the ecological environment. Therefore, the agrochemical industry is continuously searching for new insecticides. Natural products are valuable resources due to the vast biodiversity of plants and microbes. Plant-derived insecticidal molecules are secondary metabolites in plants. Generally, these secondary metabolites cause less environment pollution and are safer to natural enemies. Due to their structural diversity and biological characteristics, plant-derived natural products have received significant attention as lead compounds. Therefore, the application of these natural plant products as alternatives to synthetic insecticides has attracted more attention in recent years [1,2,3,4].

**Figure 1:**
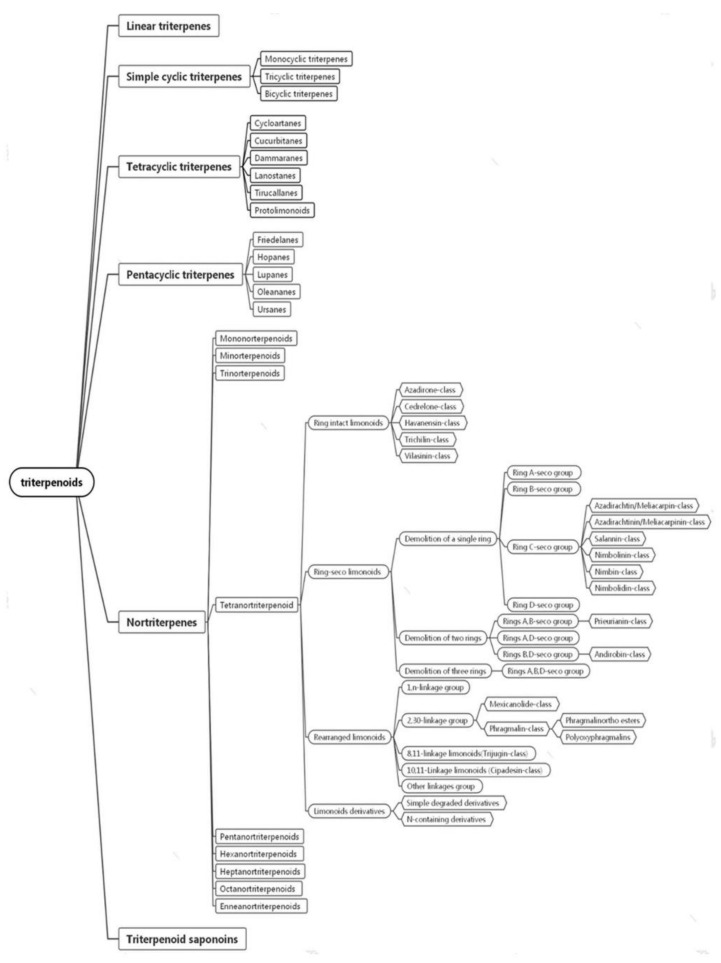
The main structural categories of triterpenes.

Triterpenes, as the main bioactive chemical compounds in Meliaceae plants, have attracted significant attention owing to their exclusive structural characteristics and remarkable biological activity. Due to their multiple bioactivities, the Meliaceae plants have been used as folk herbs in treating leprosy, eczema, asthma, malaria, fever, and pain. To date, diverse insecticidal molecules have been isolated from Meliaceae plants. A great many studies have revealed that, in these plants, triterpenoids were the active molecules [5,6]. Triterpenes are terpenoids derived from squalene, usually composed of 30 carbon atoms. The structural classification of triterpenoids is mainly grouped into six groups, including linear triterpenes, simple cyclic triterpenes (monocyclic triterpenes, bicyclic triterpenes, and tricyclic triterpenes), tetracyclic triterpenes, pentacyclic triterpenes, nortriterpenes, and triterpenoid saponins (Figure 1) [7].

This review, as a continuation of our first review (“insecticidal triterpenes in Meliaceae: plant species, molecules, and activities of eight genera (*Aglaia*, *Aphanamixis*, *Azadirachta*, *Cabralea*, *Carapa*, *Cedrela*, *Chisocheton*, and *Chukrasia*) in Meliaceae” [7]), covers naturally occurring insecticidal triterpenoids from five genera (*Cipadessa*, *Entandrophragma*, *Guarea*, *Khaya*, and *Melia*) in Meliaceae. Herein, we summarize the insecticidal plant species, insecticidal phytochemicals and their structures, various insecticidal activities, the structure–activity relationship (SAR), the insecticidal mechanism of action, and the environmental toxicity of the active insecticidal chemicals, hoping to offer some constructive information for the exploration of these chemicals as the lead compounds of novel insecticides. Furthermore, the future research perspectives are discussed.

## 2. Plant Species and Their Insecticidal Chemicals

**Figure 2:**
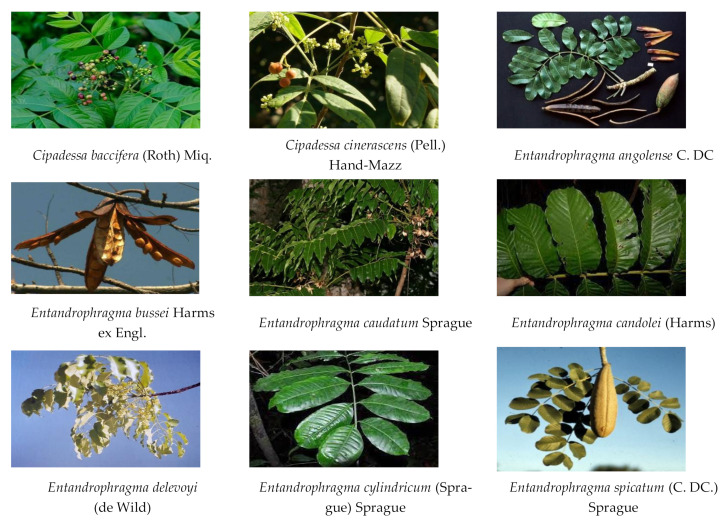

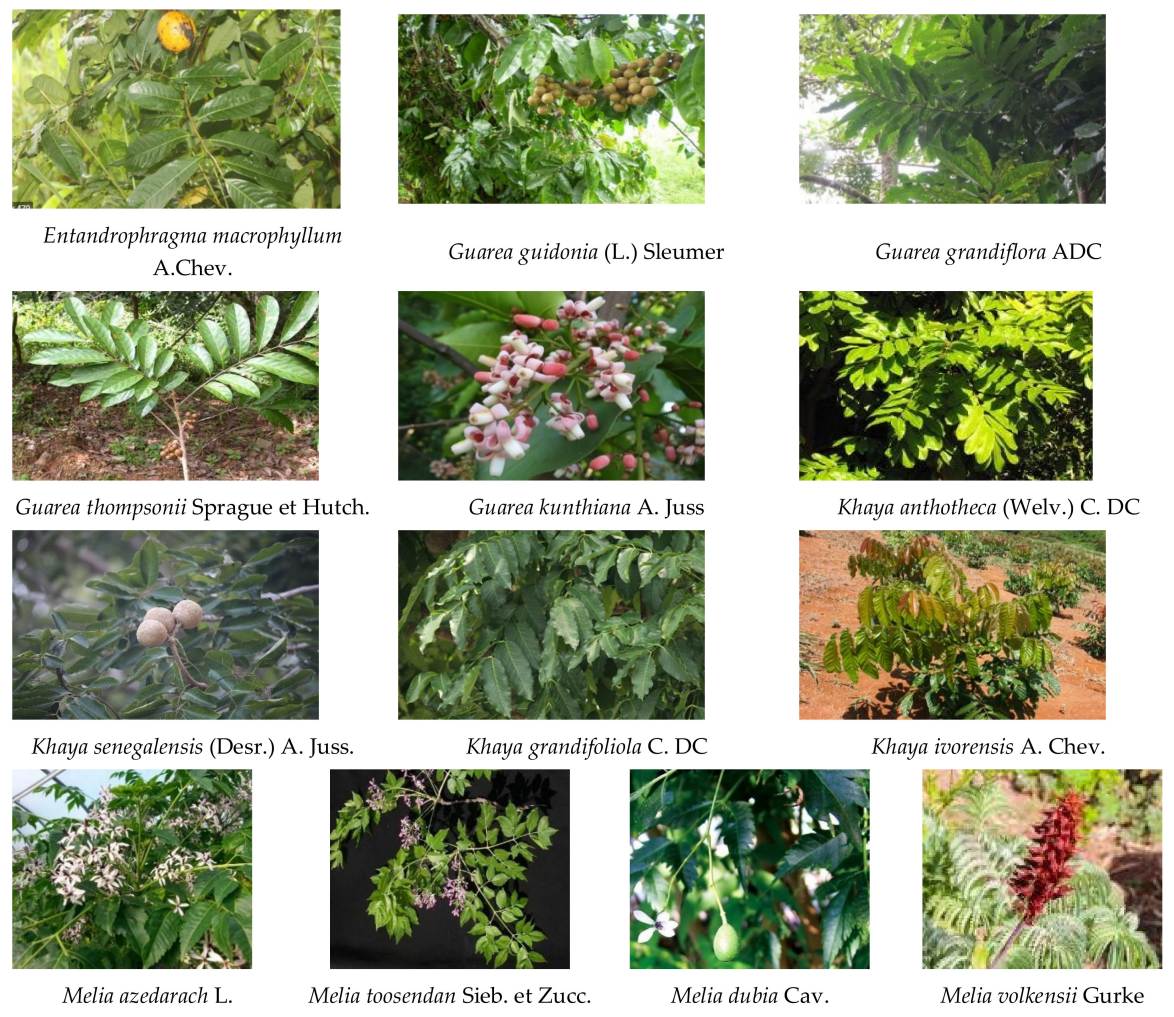
The 22 insecticidal plant species from genera *Cipadessa*, *Entandrophragma*, *Guarea*, *Khaya*, and *Melia* in Meliaceae.

In total, 22 insecticidal plant species (*Cipadessa baccifera* (Roth) Miq., *Cipadessa cinerascens* (Pell.) Hand-Mazz, *Entandrophragma angolense* C. DC., *Entandrophragma bussei* Harms ex Engl., *Entandrophragma caudatum* Sprague, *Entandrophragma candolei* (Harms), *Entandrophragma delevoyi* (de Wild), *Entandrophragma cylindricum* (Sprague) Sprague, *Entandrophragma spicatum* (C. DC.) Sprague, *Entandrophragma macrophyllum* A. Chev., *Guarea guidonia* A. Juss, *Guarea grandiflora* ADC, *Guarea thompsonii* Sprague et Hutch., *Guarea kunthiana* A. Juss, *Khaya anthotheca* (Welv.) C. DC., *Khaya senegalensis* (Desr.) A. Juss., *Khaya grandifoliola* C. DC., *Khaya ivorensis* A. Chev., *Melia azedarach* L., *Melia toosendan* Sieb. et Zucc., *Melia dubia* Cav., and *Melia volkensii* Gurke) from five genera *(Cipadessa*, *Entandrophragma*, *Guarea*, *Khaya*, and *Melia*) in Meliaceae were reported to show insecticidal activities (Table 1 and Figure 2). In these genera, the insecticidal activities of plants from *Entandrophragma* and *Melia* have attracted considerable research attention in recent years. Specifically, the insecticidal activities of plants from *Melia* have been systemically studied for several decades [8,9,10,11,12,13,14,15,16,17,18,19,20,21,22,23,24,25,26,27,28,29,30].

In all, from the aforementioned 22 plant species, 116 insecticidal chemicals were reported to be active toward 30 insect species (*Aedes aegypti* (L.), *Aphis citricidis* Kirkaldy, *Atta sexdens rubropilosa* Forel, *Brontispa longissima* (Gestro), *Callosobruchus maculatus* (Fabricius), *Cryptolestes ferrugineus* (Stephens), *Culex annulirostris* (Skuse), *Drosophila melanogaster* Meigen, *Epilachna paenulata* Germar, *Helicoverpa armigera* (Hübner), *Leptinotarsa decemlineata* (Say), *Leucania compta* Moore, *Myzus persicae* Sulzer, *Ostrinia furnacalis* Guenee, *Ostrinia nubilalis* (Hübner), *Peridroma saucia* (Hübner), *Pieris brassicae* (L.), *Pieris rapae* L., *Plutella xylostella* (L.), *Reticulitermes speratus* Kollbe, *Rhipicephalus microplus* Canestrini, *Sitophilus oryzae* L., *Spodoptera abyssinia* Guenee, *Spodoptera eridania* Cramer, *Spodoptera exigua* (Hübner), *Spodoptera frugiperda* (J. E. Smith), *Spodoptera littoralis* (Boisduval), *Spodoptera litura* (F.), *Toxoptera aurantia* (Boyer) and *Trichoplusia ni* (Hübner)). Taken together, the antifeedant activity of these plant-derived chemicals was the main studied activity [5,11,24,29,31,32,33,34,35,36,37,38,39,40,41,42,43,44,45,46]. However, the poisonous activity [5,20,25,26,41,47,48,49,50,51,52,53] and the growth-regulatory activity [18,21,23,26,32,43,50,54,55,56] have also been studied.

In summary, 80 chemicals isolated from 19 plant species (*C. baccifera*, *C. cinerascens*, *C. fruticosa*, *E. angolense*, *E. bussei*, *E. caudatum*, *E. cylindricum*, *E. delevoyi*, *E. macrophyllum*, *E. spicatum*, *G. grandiflora*, *G. thompsonii*, *K. anthotheca*, *K. grandifoliola*, *K. ivoremis*, *K. senegalensis*, *M. azedarach*, *M. toosendan*, and *M. volkensii*) in Meliaceae exhibited antifeedant activity toward 14 insect species (*D. melanogaster*, *E. paenulata*, *H. armigera*, *L. decemlineata*, *O. nubilalis*, *P. saucia*, *P. rapae*, *R. speratus*, *S. Abyssinia*, *S. eridania*, *Spodoptera exigua*, *S. frugiperda*, *S. littoralis*, and *S. litura*) (Table 2) [5,11,24,29,31,32,33,34,35,36,37,38,39,40,41,42,43,44,45,46]. Among these chemicals, the antifeedant activity of toosendanin was significant. It was reported that 0.01% toosendanin could have a 100% antifeedant effect on *S. litura*. This molecule was also effective on many other insects such as *P. rapae* and *H. armigera*. Owing to its high effect, toosendanin has been commercially applied [32,37,39,44,57,58].

Overall, 18 chemicals isolated from 17 plant species (*C. guianensis*, *C. baccifera*, *C. cinerascens*, *C. fruticosa*, *E. angolense*, *E. delevoyi*, *E. macrophyllum*, *G. grandiflora*, *G. guidonia*, *G. kunthiana*, *G. thompsonii*, *K. anthotheca*, *K. grandifoliola*, *K. ivoremis*, *K. senegalensis*, *M. azedarach*, and *M. toosendan*) in Meliaceae exhibited poisonous activity toward 10 insect species (*A. aegypti*, *A. sexdens rubropilosa*, *C. ferrugineus*, *M. persicae*, *O. furnacalis*, *P. xylostella*, *R. speratus*, *S. oryzae*, *S. frugiperda*, and *S. littoralis*) (Table 3) [5,20,25,26,41,47,48,49,50,51,52,53]. Among them, 1,3-dicinnamoyl-11-hydroxymeliacarpin, 1-cinnamoyl-3-methacryl-11-hydroxymeliacarpin, and 1-cinnamoyl-3-acetyl-11-hydroxymeliacarpin, isolated from M. azedarach, were high effective on the African cotton leafworm, *S. littoralis*, with LC_50_ values (12 days) of 2.36, 1.19, and 0.48 μg/mL, respectively [52].

Furthermore, 16 chemicals isolated from 11 plant species (*C. guianensis*, *C. baccifera*, *C. cinerascens*, *C. fruticosa*, *E. candolei*, *G. grandiflora*, *G. guidonia*, *G. kunthiana*, *K. senegalensis*, *M. azedarach*, and *M. toosendan*) in Meliaceae possessed insect growth-regulatory activity toward eight insect species (*B. longissima*, *H. armigera*, *O. furnacalis*, *O. nubilalis*, *P. saucia*, *R. microplus*, *S. frugiperda*, and *S. littoralis*) (Table 4) [18,21,23,26,32,43,50,54,55,56]. Among these chemicals, toosendanin was the most effective insect growth-regulatory agent, showing good activity toward *P. saucia*, *O. furnacalis*, *S. frugiperda*, etc. [20,23,55].

Below, we review the insecticidal plant species, the corresponding insecticidal chemicals, and their activities in detail.

### 2.1. Cipadessa

In the genus *Cipadessa*, two species, *C. baccifera* and *C. cinerascens*, have been reported to show insecticidal activities. Additionally, limonoids isolated from the leaves of *C. baccifera* showed moderate antimalarial activity [8,9].

The acetone extract of *C. baccifera* inhibited the freshly laid eggs of the mosquito *C. quinquefasciatus*. The acetone extract of the leaf of *C. baccifera* showed smoking toxicity toward mosquitoes *A. stephensi*, *A. aegypti*, and *C. quinquefasciatus* [59,60]. The hexane and dichloromethane extracts from the fruits of *C. baccifera* showed toxicity toward the leaf-cutting ant, *A. sexdens rubropilosa* [61]. Likewise, the hexane extract from the leaves of *C. baccifera* showed insecticidal activity toward the cotton bollworm, *H. armigera*. Further studies revealed that the petroleum ether extract reduced the pupation rate and pupal weight and caused a higher percentage of malformed adults. However, the hexane extract reduced the fecundity and egg hatchability in the first-generation adults [62].

A total of 12 mexicanolide limonoids and four tetranortriterpenoids were reported to show insecticidal activities. In detail, the 12 mexicanolide limonoids were cipadesin, cipadesin A, 2’*S*-cipadesin A, febrifugin, febrifugin A, 3-*O*-detigloyl-3-*O*-isobutyrylfebrifugin A, ruageanin A, khayasin, khayasin T, granatumin E, swietemahonolide, and mexicanolide. The four tetranortriterpenoids were two ring B,D-seco limonoids (cipadonoid B and cineracipadesin G) and two 10,11-linkage limonoids (cipadesin B and 3-deacetyl-cipadonoid D) [42,63,64,65,66,67].

#### 2.1.1. Mexicanolide Limonoids

In this group, 12 chemicals were reported to show insecticidal activity: cipadesin, cipadesin A, 2’*S*-cipadesin A, febrifugin, febrifugin A, 3-*O*-detigloyl-3-*O*-isobutyrylfebrifugin A, ruageanin A, khayasin, khayasin T, granatumin E, swietemahonolide, and mexicanolide [47,63,67,68,69,70].

Febrifugin A, khayasin T, cipadesin, febrifugin, ruageanin A, and cipadesin A showed poisonous activity toward the fall armyworm, *S. frugiperda*. At 50 mg/kg, the total cycle mortalities of febrifugin A and khayasin T toward the fall armyworm were 73.3% and 50%, respectively. However, the total cycle mortalities of the other four chemicals toward the fall armyworm were less than 40%. Febrifugin, khayasin T, cipadesin, and cipadesin A also showed growth-inhibitory activity toward the fall armyworm. At 50 mg/kg, febrifugin and khayasin T shortened larval phases by 1.8 and 1.2 days, respectively. At 100 mg/kg, cipadesin A and cipadesin shortened the larval phases by 2.1 and 0.8 days, respectively. Meanwhile, febrifugin also showed antifeedant activity toward the fall armyworm at 100 mg/kg [43,47,51,61,63,70,71].

Khayasin T, cipadesin, febrifugin, ruageanin A, and cipadesin A, together with two other chemicals (swietemahonolide and mexicanolide), also showed poisonous activity toward the leaf-cutting ant, *A. sexdens rubropilosa*. The median survival (S_50_) value varied from 6–9 days [42,51].

Additionally, khayasin exhibited marked insecticidal activity toward the fifth larvae of coconut leaf beetle, *B. longissimi*, with an LC_50_ value of 7.28 μg/mL at 24 h [53].

#### 2.1.2. Rings B,D-Seco Limonoids

In this group, two andirobin-type chemicals, cipadonoid B and cineracipadesin G, were reported to show insecticidal activity.

Cineracipadesin G showed antifeedant activity toward the fruit fly, *D. melanogaster*. The antifeedant index was 32.8% at 1 mM after 17 h [42]. An in vitro assay at the insect nicotinic acetylcholine receptor (nAChR) was performed for cipadonoid B, and the pI_50_ value was found to be 4.2, showing that this chemical was a weak antagonist of the insect nAChR [47,72].

#### 2.1.3. 10,11-Linkage Limonoids

In this group, two chemicals, cipadesin B and 3-deacetyl-cipadonoid D, were reported to show insecticidal activity. In detail, 3-deacetyl-cipadonoid D showed antifeedant activity toward the fruit fly, *D. melanogaster*, at 1 mM, and the antifeedant index was 39.1% after 17 h [42]. Cipadesin B was reported to show poisonous activity toward the leaf-cutting ant, *A. sexdens rubropilosa*, with a median survival (S_50_) value of 9 days [51].

### 2.2. Entandrophragma

The genus *Entandrophragma* comprises 10–12 tree species distributed exclusively in tropical Africa. In this genus, eight species, *E. angolense*, *E. bussei*, *E. caudatum*, *E. candolei*, *E. delevoyi*, *E. cylindricum*, *E. spicatum*, and *E. macrophyllum*, have been reported to show insecticidal activities [10,11,12,13,14,15,16].

In total, 16 tetranortriterpenoids were reported to show insecticidal activities. In detail, there were eight ring-seco limonoids, seven rearranged limonoids, and one ring-intact limonoid (azadirone). Furthermore, the eight ring-seco limonoids consisted of four rings A,B-seco group limonoids (prieurianin, epoxyprieurianin, prieurianin acetate, and epoxyprieurianin acetate) [32], two rings B,D-seco group limonoids (6-acetoxymethyl angolensate and methyl angolensate), one ring D-seco chemical (secomahoganin), and one ring D-seco chemical (gedunin). The seven rearranged limonoids were all 2,30-linkage group limonoids and could be further divided into two groups consisting of five mexicanolide-type limonoids (angolensin A, angolensin B, angolensin C, 3β-hydroxy-3-deoxycarapin, and xyloccensin K) and two phragmalin-type limonoids (entandrophragmin and bussein) [5,15,31,32,73,74,75].

#### 2.2.1. Ring-Seco Limonoids

In this group, eight chemicals were reported to show insecticidal activity: epoxyprieurianin, prieurianin acetate, epoxyprieurianin acetate, prieurianin, 6-acetoxymethyl angolensate, methyl angolensate, secomahoganin, and gedunin.

Rings A,B-seco group limonoids: prieurianin-type limonoids, prieurianin, epoxyprieurianin, and their acetyl derivatives, could inhibit the larval growth of the cotton bollworm, *H. armigera*. The EC_50_ values (7 days) of prieurianin and epoxyprieurianin were 18.8 and 3.2 μg/mL, respectively. In addition, the EC_50_ values (7 days) of prieurianin acetate and epoxyprieurianin acetate were 11.5 and 2.6 μg/mL, respectively [32,76].

Rings B,D-seco group limonoids: the two rings B,D-seco group limonoids, 6-acetoxymethyl angolensate and methyl angolensate, could be further classified as andirobin-class limonoids. The minimum antifeedant concentration (MAC) of 6-acetoxymethyl angolensate against the African cotton leafworm, *S. littoralis*, was 500 μg/mL [46]. Methyl angolensate showed antifeedant activity toward the tobacco cutworm, *S. litura*. At 1 μg/cm^2^, the PFI (percentage feeding index) value of methyl angolensate was 65.3 (24 h) [46].

Ring D-seco chemical: gedunin possessed various activities toward insects. It showed antifeedant activity toward the lower subterranean termite, *R. speratus*, with a PC_95_ value of 218.4 μg/disc after 30 days [77]. Gedunin also showed poisonous activity toward the fall armyworm, *S. frugiperda*, and growth-inhibitory activity toward the cotton bollworm, *H. armigera* [77,78]. In our previous review, we summarized its activity. Therefore, further information can be obtained from the review by Lin (2021) [7]. Additionally, secomahoganin showed antifeedant activity toward the African cotton leafworm, *S. littoralis*, at 1000 μg/mL [31].

#### 2.2.2. Rearranged Limonoids

In this group, seven chemicals were reported to show insecticidal activity; five of them were mexicanolide-type limonoids (angolensin A, angolensin B, angolensin C, 3β-hydroxy-3-deoxycarapin, and xyloccensin K), while two of them were phragmalin-type limonoids (entandrophragmin and bussein).

Mexicanolide-type limonoids: angolensins A–C, 3β-hydroxy-3-deoxycarapin, and xyloccensin K showed antifeedant activity toward the African cotton leafworm, *S. littoralis*. Among them, angolensins A and B showed activity at 500 μg/mL, while the others were active at 1000 μg/mL [31].

Phragmalin-class limonoids: Entandrophragmin and bussein showed feeding inhibition activity toward the European corn borer, *O. nubilalis*, at 500 μg/mL after 48 h [46].

#### 2.2.3. Ring-Intact Limonoid

Presently, in this group, only one azadirone-class chemical, azadirone, has been isolated from the genus *Entandrophragma*. This chemical showed antifeedant activity toward the Colorado potato beetle, *L. decemlineata*, with AIs (antifeedant indices) values of 11.6 ± 6.3, 22.4 ± 7.4, and 26.9 ± 5.1 at 100, 300, and 500 μg/mL (starved for 6 h and fed for 20 h) [5].

### 2.3. Guarea

In the genus *Guarea*, four species, *G. guidonia*, *G. grandiflora*, *G. thompsonii*, and *G. kunthiana*, have been reported to show insecticidal activities [17,18,19,20,21,79,80].

From these species, three tetracyclic triterpenes and five tetranortriterpenoids (three ring D-seco limonoids, one rings A,B-seco limonoid, and one rings B,D-seco limonoid) have been isolated. In detail, the three tetracyclic triterpenes included three protolimonoids (melianone, melianodiol, and 3β-*O*-tigloylmelianol). The five tetranortriterpenoids included three ring D-seco limonoids (gedunin, 7-deacetoxy-7-oxogedunin, and 6α-acetoxygedunin) [18], one rings A,B-seco limonoid (prieurianin-type chemical prieurianin) [17], and one rings B,D-seco limonoid (andirobin-class chemical methyl angolensate) [19,20,21,81].

Protolimonoid melianone showed poisonous and antifeedant activities toward the lower subterranean termite, *R. speratus*, at 100 μg/disc after 30 days. The mortality of *R. speratus* at 30 days was 95% [41]. Melianodiol showed poisonous activity toward the larvae of the mosquito *A. aegypti*. The LC_50_ value was 14.44 mg/mL and the LC_90_ value was 17.54 mg/mL after 24 h. According to the results, melianodiol could be regarded as a potential candidate for use as an ecologically sound biocontrol agent for reducing the larval population of this vector [20]. The other chemical, 3β-*O*-tigloylmelianol, was effective against the oogenesis and ecdysis of *R. (Boophilus) microplus* at concentrations of 0.01%, 0.005%, 0.0025%, and 0.00125%. After 48 h, the sexual gland index (GSI) decreased by 50% at all three concentrations [21].

At 100 μg/mL, the S_50_ values of ring D-seco type chemical 7-deacetoxy-7-oxogedunin was 9 days. It also prolonged the *S. frugiperda* larval phase by approximately 1.2 days at 50.0 mg·kg^−1^ [25,82]. Moreover, 6α-acetoxygedunin reduced the growth of the European corn borer, *O. nubilalis*, at 50 μg/mL after 20 days [18].

### 2.4. Khaya

A variety of studies have been carried out on the genus *Khaya*. In this genus, four species, *K. anthotheca*, *K. senegalensis*, *K. grandifoliola*, and *K. ivorensis*, have been reported to show insecticidal activities [22,23,24,25,26,83].

According to these reports, the ethanol extract of the stem bark of *K. ivorensis* had termiticidal activity [84]. The ethanol extracts of *K. grandifoliola* and *K. senegalensis* had ovicidal properties and larvicidal properties against the first-instar larvae of *C. maculatus* [85]. Moreover, the seed oil of *K. senegalensis* showed high potential for the control of the cowpea beetle, *C maculatus* [86]. Further research revealed that the acetone, ethanol, hexane, and methanol extracts of *K. senegalensis* also showed insecticidal activity toward the mosquito *C. annulirostris* [87]. In addition, *K. senegalensis* gum could be employed as an emulsifying agent in the formulation industry [88].

From the abovementioned plants, a total of 13 rearranged limonoids, 7 ring-seco limonoids, and 2 ring-intact limonoids were reported to show insecticidal activities. In detail, all 13 rearranged limonoids (khayanolide A, khayanolide B, khayanolide C, khayanolide D, khayanolide E, 1-*O*-acetylkhayanolide A, 1-*O*-acetylkhayanoilde B, 2-hydroxyseneganolide, khayalactol, khayanone, 6-*O*-acetylswietenolide, swietenolide, and seneganolide) were 2,30-linkage type chemicals [33,34,54]. The seven ring-seco limonoids belonged to two subgroups, namely, ring D-seco chemicals (gedunin, 7-deacetylgedunin, and 7-deacetoxy-7-oxogedunin) and rings B,D-seco chemicals (khayanoside, methyl 6-hydroxyangolensate, methyl 6-acetoxyangolensate, and methyl angolensate). Additionally, the two ring-intact limonoids included one cedrelone-type limonoid (anthothecol) and one azadirone-class chemical (azadirone) [33,34,89,90,91,92,93,94,95,96].

#### 2.4.1. Rearranged Limonoids: 2,30-Linkage Type Chemicals

In this group, 13 chemicals were reported to show insecticidal activity: khayanolide A, khayanolide B, khayanolide C, khayanolide D, khayanolide E, 1-*O*-acetylkhayanolide A, 1-*O*-acetylkhayanoilde B, 2-hydroxyseneganolide, khayalactol, khayanone, 6-*O*-acetylswietenolide, swietenolide, and seneganolid.

Among these chemicals, khayanolides A–D, 1-*O*-acetylkhayanolide A, 2-hydroxyseneganolide, and 1-*O*-acetylkhayanoilde B showed antifeedant activity toward the African cotton leafworm, *S. littoralis*. By the leaf disc method, they were active at 300, 1000, 100, 100, 200, 300, and 500 μg/mL with antifeeding activities of 21.7%, 24.8%, 57.1%, 31.4%, 38.4%, 17.3%, and 31.5%, respectively, after 6 h. In contrast, khayanolide E showed antifeedant activity toward the African cotton leafworm, *S. littoralis*, at 100 μg/mL [33,34,54]. In addition, khayanone, khayalactol, and seneganolide showed antifeeding potential with activities of 47.4%, 83.8%, and 48.0%, respectively, at 1000 μg/mL against *S. littoralis* after 6 h [34]. Studies also revealed that swietenolide and 6-*O*-acetylswietenolide possessed antifeedant activity toward *S. littoralis*. The AIs (antifeedant indices) were 94.10 ± 2.90 and 72.20 ± 19.60 at 1000 μg/mL [35]. In addition, khyanolide A, khyanolide B, 1-*O*-acetylkhayanoilde B, and khayalactol also showed growth-regulatory activity toward *S. littoralis* with EC_50_ (7 days) values of 14.65, 6.96, 16.75, and 11.48 mg/kg, respectively [54].

#### 2.4.2. Ring-Seco Limonoids

In this group, seven chemicals were reported to show insecticidal activity. Among these chemicals, three belonged to the ring D-seco group: gedunin, 7-deacetylgedunin and 7-deacetoxy-7-oxogedunin. The other four belonged to the rings B,D-seco group: khayanoside, methyl 6-hydroxyangolensate, methyl 6-acetoxyangolensate, and methyl angolensate.

The insecticidal activity of the ring D-seco group could be found in several studies [5,25,50,77,78,82,97,98,99]. These chemicals possessed more than one type of activity, and the activities were obvious. For example, gedunin showed antifeedant activity toward the lower subterranean termite, *R. speratus* (PC_95_, 113.7 μg/disc), and growth-inhibitory activity toward the cotton bollworm, *H. armigera* (EC_50_, 50.8 μg/mL), and the tobacco cutworm, *S. litura* (EC_50_, 40.4 μg/mL) [77,78]. The other two chemicals, 7-deacetylgedunin and 7-deacetoxy-7-oxogedunin, possessed insecticidal activity toward the leaf-cutting ant, *A. sexdens rubropilosa*. At 100 μg/mL, the S_50_ values of these chemicals on *A. sexdens rubropilosa* were 9 days and 11 days, respectively [24,25,82]. Further information can be obtained from the review paper by Lin [7].

Among the four rings B,D-seco group chemicals, three (methyl angolensate, methyl 6-hydroxyangolensate, and methyl 6-acetoxyangolensate) were andirobin-class chemicals. Methyl angolensate showed antifeedant and poisonous activity toward insects. It was effective at 1 μg/cm^2^ toward the tobacco cutworm, *S. litura*, with a PFI (percentage feeding index) value (24 h) of 65.3 [40,91,97,100,101,102]. Additionally, this chemical also showed poisonous activity at 50 mg/kg toward the larva of the fall armyworm, *S. frugiperda*, with a mortality rate of 40% after 7 days [50,103]. Methyl 6-acetoxyangolensate and methyl 6-hydroxyangolensate showed antifeedant activity toward the African cotton leafworm, *S. littoralis*, at 500 μg/mL using the leaf disc method after 6 h. The antifeeding activities were 23.6% and 18.0%, respectively. In contrast, khayanoside showed weaker antifeedant activity toward *S. littoralis* than methyl 6-acetoxyangolensate and methyl 6-hydroxyangolensate, and it was active at a higher concentration (1000 μg/mL) using the leaf disc method after 6 h, with an antifeeding activity of 15.1% [34].

#### 2.4.3. Ring-Intact Limonoids: Anthothecol and Azadirone

The two chemicals of this group, anthothecol and azadirone, can be further classified into two subgroups. Anthothecol is a cedrelone-type limonoid, while azadirone is an azadirone-class chemical.

The cedrelone-type limonoid anthothecol showed larvicidal activity toward the diamondback moth, *P. xylostella*. At 1 mg/mL, the mortality was 80% after 48 h [48]. At the same concentration, anthothecol also induced 60% mortality of the green peach aphid, *M. persicae*, after 48 h [48].

The azadirone-class chemical azadirone showed antifeedant activity toward the Colorado potato beetle, *L. decemlineata*, with an antifeedant index of 11.6 ± 6.3 (100 μg/mL) (starved for 6 h and fed for 20 h) [5].

### 2.5. Melia

In the genus *Melia*, four species, *M. azedarach*, *M. toosendan*, *M. dubia*, and *M. volkensii*, have been reported to show insecticidal activities [27,28,29,30,44,87,95,104,105,106,107,108,109,110,111,112,113,114,115,116,117,118,119,120,121,122,123,124,125,126,127,128,129,130,131,132,133,134,135,136,137,138,139,140,141,142,143,144,145,146,147,148,149,150,151,152,153,154,155,156,157,158,159,160,161,162,163,164,165].

**Table 2:** Antifeedant activity of insecticidal triterpenoids of plants from five genera in Meliaceae.

Compound	Plant Source	Insect	Activity	Ref.
cineracipadesin G	*Cipadessa cinerascens*	*Drosophila melanogaster*	AI = 32.8% (1 mM)	[42]
febrifugin	*Cipadessa fruticosa* *Cipadessa baccifera* *Cipadessa cinerascens*	*Spodoptera frugiperda*	AFD at 100 mg/kg	[43]
3-deacetyl-cipadonoid D	*Cipadessa cinerascens*	*Drosophila melanogaster*	AI = 39.1% (1 mM) (17 h)	[42]
angolensin A	*Entandrophragma angolense*	*Spodoptera littoralis*	AFD at 500 μg/mL	[31]
angolensin B	*Entandrophragma angolense*	*Spodoptera littoralis*	AFD at 500 μg/mL	[31]
angolensin C	*Entandrophragma angolense*	*Spodoptera littoralis*	AFD at 1000 μg/mL	[31]
3β-hydroxy-3-deoxy- carapin	*Entandrophragma angolense*	*Spodoptera littoralis*	AFD at 1000 μg/mL	[31]
xyloccensin K	*Entandrophragma angolense*	*Spodoptera littoralis*	AFD at 1000 μg/mL	[31]
entandrophragmin	*Entandrophragma cylindricum* *Entandrophragma bussei* *Entandrophragma spicatum* *Entandrophragma caudatum*	*Ostrinia nubilalis*	FI at 500 μg/mL (48 h)	[11,46,131,132]
bussein	*Entandrophragma bussei* *Entandrophragma caudatum*	*Ostrinia nubilalis*	FI at 500 μg/mL (48 h)	[11,46]
6-acetoxymethyl angolensate	*Entandrophragma angolense*	*Spodoptera littoralis*	MAC = 500 μg/mL	[11]
methyl angolensate	*Entandrophragma angolense* *Entandrophragma macrophyllum* *Guarea thompsonii* *Khaya anthotheca* *Khaya senegalensis* *Khaya grandifoliola* *Khaya ivoremis*	*Spodoptera litura*	PFI = 65.3 (24 h)	[40]
secomahoganin	*Entandrophragma angolense*	*Spodoptera littoralis*	AFD at 1000 μg/mL	[31]
azadirone	*Entandrophragma delevoyi* *Khaya anthotheca*	*Leptinotarsa decemlineata*	AI = 11.6–26.9 at 100–500 μg/mL (20 h)	[5]
gedunin	*Entandrophragma angolense* *Entandrophragma delevoyi* *Entandrophragma macrophyllum* *Guarea grandiflora* *Khaya grandifoliola*	*Reticulitermes speratus*	PC_95_ = 218.4 μg/disc (30 days)	[5,77,78]
melianone	*Guarea grandiflora*	*Reticulitermes speratus*	antifeeding activity at 100 μg/disc (30 days)	[41]
khayanolide D	*Khaya senegalensis*	*Spodoptera littoralis*	AI = 57.1 at 100 μg/mL (6 h)	[34]
khayanolide E	*Khaya senegalensis*	*Spodoptera littoralis*	MIC = 100 μg/mL	[33]
khayanolide A	*Khaya senegalensis*	*Spodoptera littoralis*	AI = 21.7 at 300 μg/mL (6 h)	[34]
khayanolide B	*Khaya senegalensis*	*Spodoptera littoralis*	AI = 24.8 at 1000 μg/mL (6 h)	[34]
2-hydroxysenega- nolide	*Khaya senegalensis*	*Spodoptera littoralis*	AI = 38.4 at 200 μg/mL (6 h)	[34]
1-*O*-acetylkhayanolide A	*Khaya senegalensis*	*Spodoptera littoralis*	AI = 31.4 at 100 μg/mL (6 h)	[34]
1-*O*-acetylkhayanoilde B	*Khaya senegalensis*	*Spodoptera littoralis*	AI = 17.3 at 300 μg/mL (6 h)	[34]
khayanolide C	*Khaya senegalensis*	*Spodoptera littoralis*	AI = 31.5 at 500 μg/mL (6 h)	[34]
khayalactol	*Khaya senegalensis*	*Spodoptera littoralis*	AI = 83.8 at 1000 μg/mL (6 h)	[34]
khayanone	*Khaya senegalensis*	*Spodoptera littoralis*	AI = 47.4 at 1000 μg/mL (6 h)	[34]
6-*O*-acetylswietenolide	*Khaya grandifoliola*	*Spodoptera littoralis*	AI = 72.2 at 1000 μg/mL	[5,35]
swietenolide	*Khaya grandifoliola*	*Spodoptera littoralis*	AI = 94.1 at 1000 μg/mL	[5,35]
seneganolide	*Khaya senegalensis*	*Spodoptera littoralis*	AI = 48.0 at 1000 μg/mL (6 h)	[34]
azadirone	*Entandrophragma delevoyi* *Khaya anthotheca*	*Leptinotarsa decemlineata*	AI = 11.6–26.9 at 100–500 μg/mL (20 h)	[5]
khayanoside	*Khaya senegalensis*	*Spodoptera littoralis*	AI = 15.1 at 1000 μg/mL (6 h)	[34]
methyl 6-hydroxyangolensate	*Khaya senegalensis*	*Spodoptera littoralis*	AI = 23.6 at 1000 μg/mL (6 h)	[34]
methyl 6-acetoxyangolensate	*Khaya senegalensis*	*Spodoptera littoralis*	AI = 18.0 at 1000 μg/mL (6 h)	[47]
meliacarpinin B	*Melia azedarach*	*Spodoptera exigua*	MIC = 50 μg/mL (2–24 h)	[36]
meliacarpinin C	*Melia azedarach*	*Spodoptera exigua*	MIC = 50 μg/mL (2–24 h)	[36]
meliacarpinin D	*Melia azedarach*	*Spodoptera exigua*	MIC = 50 μg/mL (2–24 h)	[36]
meliacarpinin A	*Melia azedarach*	*Spodoptera exigua*	MIC = 50 μg/mL (2–24 h)	[36]
salannal	*Melia toosendan*	*Pieris rapae*	AFC_50_ = 1.26 mM	[44]
3-*O*-acetylohchinolal	*Melia toosendan*	*Pieris rapae*	AFC_50_ = 0.89 mM	[44]
salannin	*Melia toosendan* *Melia azedarach*	*Pieris rapae*	AFC_50_ = 1.35 mM	[44]
		*Spodoptera eridania*	MIC = 1000 μg/mL (2–24 h)	[37]
ohchinal	*Melia toosendan*	*Pieris rapae*	AFC_50_ = 1.79 mM	[44]
nimbolinin B	*Melia toosendan*	*Spodoptera eridania*	MIC = 1000 μg/mL (2–24 h)	[37]
toosendanin	*Melia azedarach* *Melia toosendan*	*Spodoptera eridania*	MIC = 300 μg/mL (2–24 h)	[5,32,37,39,44,57,58]
		*Pieris rapae*	AFC_50_ = 0.21 mM	
		*Peridroma saucia*	DC_50_ = 8.04 μg/cm^2^	
		*Helicoverpa armigera*	FI_50_ = 56.6 μg/mL (6 h)	
		*Epilachna paenulata*	ED_50_ = 3.69 μg/cm^2^ (24 h)	
		*Spodoptera littoralis*	AFC_50_ = 200 μg/mL	
		*Spodoptera litura*	100% antifeedant rate at 0.01% toosendanin	
		*Spodoptera abyssinia*	76.5% antifeedant rate at 0.1% toosendanin	
nimbolidin C	*Melia toosendan*	*Spodoptera eridania*	MIC = 500 μg/mL (2–24 h)	[37]
nimbolidin D	*Melia toosendan*	*Spodoptera eridania*	MIC = 500 μg/mL (2–24 h)	[37]
nimbolidin E	*Melia toosendan*	*Spodoptera eridania*	MIC = 500μg/mL (2–24 h)	[37]
nimbolidin F	*Melia toosendan*	*Spodoptera eridania*	MIC = 500 μg/mL (2–24 h)	[37]
3-*O*-acetylohchinolal	*Melia toosendan*	*Spodoptera eridania*	MIC = 1000 μg/mL (2–24 h)	[37]
ohchinolide C	*Melia toosendan*	*Spodoptera eridania*	MIC = 1000 μg/mL (2–24 h)	[37]
volkensin	*Melia volkensii*	*Spodoptera frugiperda*	ED_50_ = 3.5 μg/cm^2^ (15 h)	[29]
hydroxylactone	*Melia volkensii*	*Spodoptera frugiperda*	ED_50_ = 6 μg/cm^2^ (15 h)	[29]
6-acetylsendanal	*Melia toosendan*	*Pieris rapae*	AFC_50_ = 1.32 mM	[44]
iso-chuanliansu	*Melia toosendan* *Melia toosendan*	*Pieris rapae*	AFC_50_ = 0.46 mM	[44]
		*Spodoptera littoralis*	MIC = 300 μg/mL (2–24 h)	[29]
amoorastatone	*Melia toosendan*	*Pieris rapae*	AFC_50_ = 0.63 mM	[44]
12-hydroxyamoorastatone	*Melia toosendan*	*Pieris rapae*	AFC_50_ = 0.64 mM	[44]
mesendanin H	*Melia toosendan*	*Pieris rapae*	AFC_50_ = 0.11 mM	[44]
meliatoosenin E	*Melia toosendan*	*Pieris rapae*	AFC_50_ = 1.03 mM	[44]
trichilin B	*Melia azedarach*	*Spodoptera exigua*	MIC = 200 μg/mL (6–24 h)	[36]
aphanastatin	*Melia azedarach*	*Spodoptera exigua*	MIC = 200 μg/mL (6–24 h)	[36]
azedarachin A	*Melia azedarach*	*Spodoptera exigua*	MIC = 200 μg/mL (6–24 h)	[36]
12-*O*-acetyltrichilin B	*Melia azedarach*	*Spodoptera exigua*	MIC = 400 μg/mL (6–24 h)	[36]
		*Spodoptera eridania*	MIC = 400 μg/mL (6–24 h)	[37]
1,12-di-*O*-acetyltrichilin B	*Melia azedarach*	*Spodoptera exigua*	MIC = 400 μg/mL (6–24 h)	[36]
trichilin H	*Melia azedarach*	*Spodoptera exigua*	MIC = 400 μg/mL (6–24 h)	[36]
trichilin D	*Melia azedarach*	*Spodoptera exigua*	MIC = 400 μg/mL (6–24 h)	[36]
meliatoxin A_2_	*Melia azedarach*	*Spodoptera exigua*	MIC = 400 μg/mL (6–24 h)	[36]
12-*O*-acetylazedarachin A	*Melia azedarach*	*Spodoptera exigua*	MIC = 400 μg/mL (6–24 h)	[36]
12-*O*-acetylazedarachin B	*Melia azedarach*	*Spodoptera exigua*	MIC = 400 μg/mL (6–24 h)	[36]
azedarachin C	*Melia azedarach*	*Spodoptera exigua*	MIC = 400 μg/mL (6–24 h)	[36]
trichilin I	*Melia toosendan*	*Spodoptera eridania*	MIC = 400 μg/mL (2–24 h)	[37]
trichilin J	*Melia toosendan*	*Spodoptera eridania*	MIC = 400 μg/mL (2–24 h)	[37]
trichilin K	*Melia toosendan*	*Spodoptera eridania*	MIC = 400 μg/mL (2–24 h)	[37]
trichilin L	*Melia toosendan*	*Spodoptera eridania*	MIC = 400 μg/mL (2–24 h)	[37]
12-deacetyltoosendanin	*Melia toosendan*	*Spodoptera eridania*	MIC = 150 μg/mL (2–24 h)	[37]
		*Spodoptera littoralis*	MIC = 250 μg/mL (2–24 h)	[29]
l-*O*-acetyltrichilin H	*Melia toosendan*	*Spodoptera littoralis*	MIC = 300 μg/mL (2–24 h)	[29]
neoazedarachin A	*Melia toosendan*	*Spodoptera littoralis*	MIC = 400 μg/mL (2–24 h)	[29]
neoazedarachin B	*Melia toosendan*	*Spodoptera littoralis*	MIC = 400 μg/mL (2–24 h)	[29]
neoazedarachin D	*Melia toosendan*	*Spodoptera littoralis*	MIC = 400 μg/mL (2–24 h)	[29]
meliartenin	*Melia azedarach*	*Epilachna paenulata*	ED_50_ = 0.80 µg/cm^2^ (24 h)	[39]
12-hydroxia-moorastatin	*Melia azedarach*	*Epilachna paenulata*	ED_50_ = 0.80 µg/cm^2^ (24 h)	[39]
1-cinnamoyltrichilinin	*Melia volkensii*	*Spodoptera littoralis*	MAC = 1000 μg/mL	[45]

DC_50_: concentration that deters feeding of fourth-instar larvae by 50%; ED_50_ is the dosage required to give an antifeedant index of 50%; AI: antifeedant index; MIC: minimum inhibitory concentration; AFC_50_: median antifeeding concentration; FI_50_: dietary concentration showing 50% feeding inhibition; MAC: minimum antifeedant concentration.

From this genus, a total of 30 ring-intact limonoids and 19 ring-seco limonoids were reported to show insecticidal activities. In detail, the 30 ring-intact limonoids included 29 trichilin-type limonoids and one havanensin-class limonoid (6-acetylsendanal). Specifically, the 29 trichilin-type limonoids were toosendanin, 12-deacetyltoosendanin, trichilin B, trichilin D, trichilin H, trichilin I, trichilin J, trichilin K, trichilin L, 1, 12- di-*O*-acetyltrichilin B, 12-*O*-acetyltrichilin B, l-*O*-acetyltrichilin H, 1-cinnamoyltrichilinin, azedarachin A, azedarachin C, 12-*O*-acetylazedarachin A, 12-*O*-acetylazedarachin B, neoazedarachin A, neoazedarachin B, neoazedarachin D, meliartenin, meliatoosenin E, meliatoxin A_2_, mesendanin H, amoorastatone, 12-hydroxyamoorastatone, 12-hydroxiamoorastatin, aphanastatin, and iso-chuanliansu. The 19 ring C-seco limonoids were meliacarpinin B, meliacarpinin C, meliacarpinin D, meliacarpinin A, 1,3-dicinnamoyl-11-hydroxymeliacarpin, 1-cinnamoyl-3-methacryl-11-hydroxymeliacarpin, 1-cinnamoyl-3- acetyl-11-hydroxymeliacarpin, salannin, salannal, ohchinal, 3-*O*-acetylohchinolal, nimbolinin B, nimbolidin C, nimbolidin D, nimbolidin E, nimbolidin F, ohchinolide C, volkensin, and hydroxylactone [29,36,37,38,39,44,166,167,168,169,170,171,172,173,174,175,176,177,178,179,180,181,182,183,184,185,186,187,188,189].

#### 2.5.1. Ring-Intact Limonoids

The most-studied chemical in this group was toosendanin. Toosendanin is a trichilin-class limonoid isolated from *M. toosendan* and *M. azedarach* [37,49]. The principle bioactive chemicals in *M. toosendan* are toosendanin-type limonoids known as tetranortriterpenoids and intact-ring protolimonoids, which are euphane- or tirucallane-type triterpenoids. It is believed that toosendanin and its derivatives are formed by the loss of four carbons from the side chain of the euphane (20*R*) or tirucallane (20*S*) skeleton, which then cyclize to form the 17β-furan ring [5].

Toosendanin and its derivatives demonstrate high insecticidal activity and are important insecticidal molecules derived from plants. In China, toosendanin is used as an important Chinese traditional insecticide and has been registered and commercialized. Studies on the insecticidal activity of various formulations of toosendanin and residue analysis using IC-ELISA can be easily found. The studied formulations include 2% toosendanin EW, 2% toosendanin ME, 2% toosendanin SL, and 2% toosendanin WP [190,191].

Toosendanin has shown various activities including antifeeding, deterring, growth-inhibitory, contact poisoning, and stomach poisoning activities [5,37,44,49,54,57,175,192,193,194,195,196]. Specially, toosendanin has marked systemic properties. It could control the newly hatched larvae of the rice yellow stem borer, *T. incertulas*, inside of the rice stem [195].

**Table 3:** Poisonous activity of insecticidal triterpenoids of plants from five genera in Meliaceae.

Compound	Plant Source	Insect	Activity	Ref.
cipadesin	*Cipadessa fruticosa* *Cipadessa baccifera*	*Spodoptera frugiperda*	S_50_ = 7 days at 100 μg/mL	[51]
febrifugin	*Cipadessa fruticosa* *Cipadessa baccifera* *Cipadessa cinerascens*	*Spodoptera frugiperda*	S_50_ = 6 days at 100 μg/mL	[51]
ruageanin A	*Cipadessa fruticosa*	*Atta sexdens rubropilosa*	S_50_ = 6 days at 100 μg/mL	[51]
swietemahonolide	*Cipadessa fruticosa*	*Atta sexdens rubropilosa*	S_50_ = 8 days at 100 μg/mL	[51]
mexicanolide	*Cipadessa fruticosa*	*Atta sexdens rubropilosa*	S_50_ = 6 days at 100 μg/mL	[51]
cipadesin A	*Cipadessa fruticosa* *Cipadessa baccifera* *Cipadessa cinerascens*	*Spodoptera frugiperda*	MR: less than 40% at 50 mg/kg	[47]
febrifugin A	*Cipadessa fruticosa*	*Spodoptera frugiperda*	MR: 73.3% at 50 mg/kg	[47]
khayasin T	*Cipadessa fruticosa* *Cipadessa baccifera*	*Spodoptera frugiperda*	MR: 50% at 50 mg/kg	[47]
melianodiol	*Guarea grandiflora* *Guarea kunthiana*	*Aedes aegypti*	LC_50_ = 14.44 μg/mL LC_90_ = 17.54 μg/mL (24 h)	[20]
melianone	*Guarea grandiflora*	*Reticulitermes speratus*	MR: 95% at 100 μg/disc (30 days)	[41]
gedunin	*Entandrophragma angolense* *Entandrophragma delevoyi* *Entandrophragma macrophyllum* *Guarea grandiflora* *Khaya grandifoliola*	*Spodoptera frugiperda*	LM: LC_50_ = 39 μg/mL (30 days)	[5,77,78]
methyl angolensate	*Entandrophragma angolense* *Entandrophragma macrophyllum* *Guarea thompsonii* *Khaya anthotheca* *Khaya senegalensis* *Khaya grandifoliola* *Khaya ivoremis*	*Spodoptera frugiperda*	MR: 40% at 50 mg/kg (7 days)	[50]
7-deacetoxy-7-oxo -gedunin	*Guarea grandiflora* *Guarea guidonia* *Carapa guianensis*	*Atta sexdens rubropilosa*	S_50_ = 9 days at 100 μg/mL	[25]
anthothecol	*Khaya anthotheca*	*Plutella xylostella*	MR: 30–80% at 0.25–1 mg/mL (48 h)	[48]
		*Myzus persicae*	MR: 30%, 60% at 0.5, 1 mg/mL (48 h)	
toosendanin	*Melia azedarach* *Melia toosendan*	*Sitophilus oryzae*	LC_50_ = 675 μg/mL (6 weeks)	[5,49,195]
		*Cryptolestes ferrugineus*	LC_50_ = 1875 μg/mL (6 weeks)	
		*Ostrinia furnacalis*	Mortality: 58.33% at 0.4 μg (average 3.1 days)	
		*Spodoptera frugiperda*	LC_50_ = 7.0 μg/mL	
1,3-dicinnamoyl-11- hydroxymeliacarpin	*Melia azedarach*	*Spodoptera littoralis*	LC_50_ = 2.36 μg/mL (12 days)	[52]
1-cinnamoyl-3- methacryl-11-hydroxy-meliacarpin	*Melia azedarach*	*Spodoptera littoralis*	LC_50_ = 1.19 μg/mL (12 days)	[52]
1-cinnamoyl-3-acetyl-11-hydroxymeliacarpin	*Melia azedarach*	*Spodoptera littoralis*	LC_50_ = 0.48 μg/mL (12 days)	[52]
2’S-cipadesin A	*Cipadessa baccifera*	AChE	inhibitory activity (AChE) at 50 mM	[47]
granatumin E	*Cipadessa baccifera*	AChE	inhibitory activity (AChE) at 50 mM	[47]
3-*O*-detigloyl-3-*O*-isobutyrylfebrifugin A	*Cipadessa baccifera*	AChE	inhibitory activity (AChE) at 50 mM	[47]
cipadonoid B	*Cipadessa baccifera* *Cipadessa cinerascens*	insect nAChR	pI_50_ = 4.2	[47]
khayasin	*Cipadessa baccifera*	*Brontispa longissima*	LC_50_ = 7.28 μg/mL (24 h)	[53]

S_50_: survival median; MR: mortality rate; LM: larval mortality; LC_50_: median lethal concentration.

**Table 4:** Growth regulatory activity of insecticidal triterpenoids of plants from five genera in Meliaceae.

Compound	Plant Source	Insect	Activity	Ref.
cipadesin	*Cipadessafruticosa* *Cipadessa baccifera*	*Spodoptera frugiperda*	LPE: 0.8 days	[43]
febrifugin	*Cipadessa fruticosa* *Cipadessa baccifera* *Cipadessa cinerascens*	*Spodoptera frugiperda*	LPE: 1.8 days	[43]
khayasin T	*Cipadessa fruticosa* *Cipadessa baccifera*	*Spodoptera frugiperda*	LPE: 1.2 days	[43]
cipadesin A	*Cipadessa fruticosa* *Cipadessa baccifera* *Cipadessa cinerascens*	*Spodoptera frugiperda*	LPE: 2.1 days	[43]
prieurianin	*Entandrophragma candolei* *Guarea guidonia*	*Helicoverpa armigera*	GIL, EC_50_ = 18.8 μg/mL (7 days)	[32]
epoxyprieurianin	*Entandrophragma candolei*	*Helicoverpa armigera*	GIL, EC_50_ = 3.2 μg/mL (7 days)	[32]
prieurianin acetate	*Entandrophragma candolei*	*Helicoverpa armigera*	GIL, EC_50_ = 11.5 μg/mL (7 days)	[32]
epoxyprieurianin acetate	*Entandrophragma candolei*	*Helicoverpa armigera*	GIL, EC_50_ = 2.6 μg/mL (7 days)	[32]
6α-acetoxy-gedunin	*Guarea kunthiana* *Guarea grandiflora*	*Ostrinia nubilalis*	Reduced growth at 50 μg/mL	[18,56]
3β-*O*-tigloylmelianol	*Guarea kunthiana*	*Rhipicephalus microplus*	GSI reduced 50% (48 h)	[21]
7-deacetoxy-7-oxogedunin	*Guarea grandiflora* *Guarea guidonia* *Carapa guianensis*	*Spodoptera frugiperda*	LPE: 1.2 days	[50]
1-*O*-acetylkhayanoilde B	*Khaya senegalensis*	*Spodoptera littoralis*	GI, EC_50_ = 16.75 mg/kg (7 days)	[54]
khyanolide A	*Khaya senegalensis*	*Spodoptera littoralis*	GI, EC_50_ = 14.65 mg/kg (7 days)	[54]
khyanolide B	*Khaya senegalensis*	*Spodoptera littoralis*	GI, EC_50_ = 6.96 mg/kg (7 days)	[54]
khayalactol	*Khaya senegalensis*	*Spodoptera littoralis*	GI, EC_50_ = 11.48 mg/kg (7 days)	[54]
toosendanin	*Melia azedarach* *Melia toosendan*	*Peridroma saucia*	EC_50_ = 42.3 μg/mL after 7 days	[55,175,194]
		*Ostrinia furnacalis*	Inhibition of body weight and pupation	
		*Spodoptera frugiperda*	Inhibition of body weight	

EC_50_: concentration reducing growth by 50% relative to controls; LPE: larval phase extended; GIL: inhibition of larval growth; GSI: sexual gland index; GI: growth inhibition.

Toosendanin showed strong antifeedant activity toward insects such as *P. rapae*, *O. furnacalis*, *P. saucia*, *S. incertulas*, *O. furnacalis*, *L. compta*, *P. xylostrella*, *S. litura*, *A. citricidis*, and *T. aurantia* [5,32,37,44,57,58]. Toosendanin at the concentration of 0.01% could result in a 100% antifeedant rate against the tobacco cutworm, *S. litura*, while toosendanin at the concentration of 0.1% could result in an antifeedant rate of 76.5% against the lawn caterpillar, *S. abyssinia* [196]. Using the leaf disc choice test, the DC_50_ value (concentration deterring feeding by 50%) was 8.04 μg/cm^2^ against the fourth-instar larvae of the variegated cutworm, *P. saucia* [57]. On the cotton bollworm, *H. armigera*, the EC_50_ value (concentration inhibiting larval growth by 50% relative to controls) of toosendanin was 26.8 μg/mL 7 days after the treatment. The FI_50_ (dietary concentration showing 50% feeding inhibition) value for toosendanin on the third-instar larvae of the cotton bollworm, *H. armigera*, was 56.6 μg/mL [32]. As for the ladybird beetle, *E. paenulata*, the ED_50_ value of toosendanin was 3.69 μg/cm^2^ after 24 h [100]. It was also effective against the third-instar larvae of the African cotton leafworm, *S. littoralis*, at 200 μg/mL [5]. Other studies showed that the MIC value of toosendanin was 300 μg/mL against the southern armyworm, *S. eridania*, in 2–24 h [37].

Meanwhile, toosendanin also exhibited poisonous activity and insect growth-inhibitory activity. For example, toosendanin showed growth-inhibitory effects on the variegated cutworm, *P. saucia*, with an EC_50_ value of 42.3 μg/mL 7 days after treatment [55]. When tested at a concentration of 0.05%, toosendanin could inhibit the body weight of the Asian corn borer, *O. furnacalis*, by 61.52% after 2 days of treatment [195]. Toosendanin possessed obvious poisonous activity toward the fall armyworm, *S. frugiperda*, with an LC_50_ value of 7.0 μg/mL [5]. It also showed poisonous activity toward the rice weevil, *S. oryzae*, and the rusty grain beetle, *C. ferrugineus*. After 6 weeks, the LC_50_ values of toosendanin on them were 675 and 1875 μg/mL, respectively [49]. Another study showed that, when tested using the method of topical application, 0.4 μg of toosendanin resulted in a mortality of 58.33% at an average of 3.1 days on the Asian corn borer, *O. furnacalis* [194]. Furthermore, toosendanin, at 10 μg/mL, could deter the oviposition activity of the cabbage looper, *T. ni*, and the diamondback moth [139].

The meliacin-type limonoids 6-acetylsendanal, iso-chuanliansu, amoorastatone, 12-hydroxyamoorastatone, mesendanin H, and meliatoosenin E showed antifeedant activities toward the fifth-instar larvae of the cabbage worm, *P. rapae*, with AFC_50_ values of 1.32, 0.46, 0.63, 0.64, 0.11, and 1.03 mM, respectively. In contrast, the AFC_50_ value of toosendanin was 0.21 mM [36].

The minimum inhibitory concentrations (MICs), reflecting the antifeeding activity of trichilin-type limonoids against different insects, for trichilin B, aphanastatin, azedarachin A, 12-*O*-acetyltrichilin B, 1,12-di-*O*-acetyltrichilin B, trichilin H, trichilin D, meliatoxin A_2_, 12-*O*-acetylazedarachin A, 12-*O*-acetylazedarachin B, and azedarachin C ranged from 200–400 μg/mL against *S. exigua* in 6–24 h [36]. For *S. eridania*, the MICs of 12-*O*-acetyltrichilin B, trichilin I, trichilin J, trichilin K, trichilin L, and 12-deacetyltoosendanin ranged from 150–400 μg/mL in 2–24 h. In contrast, the MIC of toosendanin was 300 μg/mL. It is noteworthy that the MIC of 12-deacetyltoosendanin was 150 μg/mL, which was lower than that of toosendanin [37]. More studies on *S. littoralis* showed that the MICs of l-*O*-acetyltrichilin H, neoazedarachin A, neoazedarachin B, neoazedarachin D, 12-deacetyltoosendanin, and iso-chuanliansu varied from 250–400 μg/mL in 2–24 h [38].

The other two trichilin-type limonoids, meliartenin and 12-hydroxiamoorastatin, were interchangeable isomers and could inhibit the feeding activity of *E. paenulata*, with an ED_50_ value of 0.80 µg/cm^2^ (24 h). They also showed poisonous activity, with an LD_50_ value of 0.76 µg/cm^2^ at 96 h [110]. Furthermore, the trichilin-class limonoid 1-cinnamoyltrichilinin showed antifeedant activity toward *S. littoralis* with a minimum antifeedant concentration (MAC) value of 1000 mg/L [45].

#### 2.5.2. Ring C-Seco Limonoids

The azadirachtinin/meliacarpinin-class chemicals meliacarpinin A, meliacarpinin B, meliacarpinin C, and meliacarpinin D showed growth-inhibitory activity toward the beet armyworm, *S. exigua*, with a minimum inhibitory concentration (MIC) of 50 μg/mL (6–24 h) [36]. The azadirachtin/meliacarpin-class limonoids 1,3-dicinnamoyl-11-hydroxymeliacarpin, 1-cinnamoyl-3-methacryl-11-hydroxymeliacarpin, and 1-cinnamoyl-3-acetyl-11-hydroxymeliacarpin showed poisonous activity toward the neonate larvae of the cotton leafworm, *S. littoralis*, with LC_50_ values (12 days) of 2.36, 1.19, and 0.48 μg/mL, respectively [52].

The salannin-class limonoids salannal, 3-*O*-acetylohchinolal, salannin, and ohchinal showed antifeedant activities toward the fifth-instar larvae of the cabbage worm, *P. rapae*, with AFC_50_ values of 1.26, 0.89, 1.35, and 1.79 mM, respectively [83]. However, when compared with toosendanin, salannin showed a relatively weaker antifeedant activity, with an MIC of 1000 μg/mL, toward the southern armyworm, *S. eridania*, in 2–24 h (compared to 300 μg/mL for toosendanin) [37].

The MICs of nimbolidin-type nimbolidin C, nimbolidin D, nimbolidin E, nimbolidin F, nimbin-type chemical 3-*O*-acetylohchinolal, and nimbolinin-type chemical ohchinolide C were 500, 500, 500, 500, 1000, and 1000 μg/mL, respectively, against *S. eridania* in 2–24 h [37].

In addition, when compared with toosendanin, the nimbolinin-type chemical nimbolinin B showed a relatively weaker antifeedant activity toward *S. eridania* with an MIC of 1000 μg/mL in 2–24 h (compared to 300 μg/mL for toosendanin) [37]. Furthermore, studies on nimbolinin-type chemicals volkensin and hydroxylactone (isolated from *M. volkensii*) revealed that they were antifeedant agents against the third-instar larvae of the fall armyworm, *S. frugiperda*. In the choice assays using corn leaf discs, the ED_50_ values of the two molecules were 3.5 and 6 μg/cm^2^, respectively (15 h) [29].

## 3. Structures and Structure–Activity Relationship (SAR) of the Insecticidal Chemicals

### 3.1. Structures of the Insecticidal Chemicals

A total of 116 insecticidal chemicals were summarized, including 34 ring-intact limonoids, 31 ring-seco limonoids, 48 rearranged limonoids, and 3 tetracyclic triterpenes. The structures of the chemicals are shown in Figure 3, Figure 4, Figure 5, Figure 6, Figure 7, Figure 8, Figure 9, Figure 10, Figure 11, Figure 12, Figure 13, Figure 14, Figure 15, Figure 16, Figure 17, Figure 18 and Figure 19.

The 34 ring-intact limonoids included 29 trichilin-class chemicals, 3 azadirone-class chemicals, 1 cedrelone-class, and 1 havanensin-class limonoid. The 31 ring-seco limonoids consisted of 16 ring C-seco group chemicals, 8 rings B,D-seco group chemicals, 4 rings A,B-seco group chemicals, and 3 ring D-seco group chemicals. Furthermore, among the 48 rearranged limonoids, 46 were 2,30-linkage group chemicals and 2 were 10,11-linkage group chemicals. Specifically, the 46 chemicals belonging to the 2,30-linkage group could be subdivided into 24 mexicanolide-class chemicals and 22 phragmalin-class chemicals. Additionally, the three tetracyclic triterpenes were protolimonoids.

### 3.2. Structure–Activity Relationship (SAR) of the Insecticidal Chemicals

Structure–activity relationship (SAR) or quantitative structure–activity relationship (QSAR) analysis can be used for the rational design of novel drugs and pesticides. Substantial efforts have been dedicated to these issues [197,198,199]. Among these 116 chemicals, the SARs of toosendanin, khayanolide B, 1-*O*-acetylkhayanolide B, febrifugin, and melianodiol have been studied [20,43,45,47,54,200,201,202].

In continuation of the program aimed at the discovery and development of natural product-based insecticidal agents, according to the insecticidal activity of 12 semi-synthesized 28-acyloxy derivatives of toosendanin (2a–l) against the pre-third-instar larvae of the rice ear-cutting caterpillar, *M. separata*, in vivo at the concentration of 1 mg/mL, Xu et al. (2011) concluded that the butanoyloxy or phenylacryloyloxy moiety at the 28-position of toosendanin was essential for insecticidal activity [200]. Furthermore, Zhang et al. (2013) synthesized 18 alkyl/alkenylacyloxy derivatives at the C-28 position adopting the exo-configuration of toosendanin (3a–r) via the reaction of toosendanin with fatty acids in the presence of *N*,*N*′-diisopropylcarbodiimide and 4-dimethylaminopyridine. The activity of these 18 molecules tested on the pre-third-instar larvae of the rice ear-cutting caterpillar, *M. separata*, revealed that compounds 3e and 3o displayed more promising insecticidal activity than their natural precursor, toosendanin. It was revealed that, for the *n*-alkyloyloxy series derivatives, the proper length of the side chain R at the C-28 position of toosendanin was very important for insecticidal activity [201]. Another structure–activity relationship study of toosendanin derivatives indicated that the sites around R4 and R5 also contributed to the activity [202].

Khayanolide B and 1-*O*-acetylkhayanolide B with a C2–C14 ether linkage and hydroxyl group at C8 were more potent antifeedants than khayanolide A with a C8–C14 epoxide group and a keto-carbonyl group at C-2. The presence of a hydroxyl group at C-1 slightly enhanced the antifeedant activity of khayanolide B compared with the acetoxy group in 1-*O*-acetylkhayanolide B, indicating that the substituents at C-1 in this type of molecule had no marked effect on antifeedant activity [54].

Analysis revealed that febrifugin A had a furan-ring oxygenated group at C-21 and a hydroxy group at C-23, which contributed to the insecticidal activity. The high insecticidal activity of febrifugin A further confirmed that the hydroxyl group on C-23 and the carbonyl group on C-21 had a great influence on the activity. Compared with the furan ring oxygenated at C-21 and C-23, intact and seco-rings and an intact furan ring in the limonoids showed more significant antifeedant activity [43,47].

For the protolimonoid melianodiol, the presence of a carbonyl moiety at C-3 in the 21,23-epoxy-21,24,25-trihydroxy-tirucall-7-ene-type skeleton played an important role in the insecticidal activity [20]. Additionally, the 12α-OH function of trichilin-class limonoids was the most potent, followed by 12β-OH, 12-desoxy, and 12α-acetoxy groups in order of decreasing potency [45].

## 4. Insecticidal Mechanism of Action

The study of the insecticidal mechanism of action (MOA) of triterpenoids from these five genera mainly focused on the MOA of toosendanin. Several MOA studies of other molecules reported on the inhibition of certain enzymes. For example, khayanolide B was reported to show weak inhibitory activities toward the enzymes acetylcholinesterase (AChE), butyrycholinesterase (BuChE), and lipoxygenase (LOX) in a concentration-dependent manner [203]. The inhibition of AChE by the mexicanolide limonoids 3-*O*-detigloyl-3-*O*-isobutyrylfebrifugin A, granatumin E, khaysin T, and 2’S-cipadesin A have also been reported, and they showed moderate inhibitory activities against AChE at 50 mM [37]. In addition, prieurianin was reported as an antagonist of 20-hydroxyecdysone. When tested with the *D. melanogaster* B-II cell line, the ED_50_ value of prieurianin was 10^−5^ M with a 20-hydroxyecdysone concentration of 5 × 10^−8^ M [204].

The MOA of toosendanin has been systemically studied. Using the electrophysiological technique, the mechanism study of toosendanin as a feeding deterrent for the larvae of the cabbage butterfly, *P. brassicae*, demonstrated that toosendanin stimulated a deterrent receptor cell located in the medial maxillary sensillum styloconicum. Toosendanin, even at the low concentration of 10^−9^ M, also inhibited the responses of both the sugar and the glucosinolate receptor cell localized in the lateral sensillum styloconicum, in a dose-dependent manner. However, the taste neurons responding to amino acids or deterrents in the lateral sensillum were not affected by toosendanin. Therefore, it could be concluded that the sensory code underlying feeding behavior was modulated by toosendanin via several different peripheral sensory mechanisms [205]. Further studies showed that toosendanin seemed to specifically induce feeding deterrence in the larvae of the cotton bollworm, *H. armigera*, and apparently stimulated deterrent receptor cells and reduced neural input from taste cells specialized to detect feeding stimulants [30].

The possible mechanism underlying the poisonous activity of toosendanin has also been analyzed. It was found that the activities of protease and microsome multifunctional oxidase (MFO) in the midgut tissue of the larvae of the cabbage worm, *P. rapae*, fed with toosendanin were inhibited. However, the activities of lipase, amylase, and acetylcholinesterase were not significantly affected. The physiological metabolism of the larvae was disturbed, and abnormal biological oxidation was carried out in the body, while the metabolic level decreased. Histological observation revealed degradation in the microvilli, hyperplasia of the smooth endoplasmic reticulum, and condensation of chromatin. Moreover, immunohistochemical analysis revealed that gold particles existed on the microvilli of columnar cells and goblet cells, and they gradually accumulated with the exacerbation of poisoning symptoms, showing that toosendanin targeted the microvilli of midgut cells. In addition, it inhibited the central nervous system of the larvae [206,207,208,209].

Furthermore, studies on the larvae and female adults of the mosquito, *A. aegypti*, revealed that topical application or ingested toosendanin dose-dependently disrupted yolk deposition in oocytes, blood ingestion and digestion, and ovary ecdysteroid production in blood-fed females [210]. It is noteworthy that medicinal studies demonstrated that toosendanin selectively affected neurotransmitter release, effectively antagonized botulism, induced cell differentiation and apoptosis, inhibited proliferation of various human cancer cells, and inhibited K^+^-channel and facilitated L-type Ca^2+^-channel activity [211]. These results are good starting points for further research on the MOA of toosendanin as an insecticidal molecule.

## 5. Environmental Toxicity

In fact, various extracts of plants or some pure chemicals in Meliaceae have been used as traditional medicines. The ethnomedical uses of the plant are as varied as the different cultures and geographical people that make use of the plant. For example, the stem bark of *K. senegalensis* has been used in the treatment of several conditions, including stomach pain, malaria, fever, and blennorrhagia, in Africa [212]. The pure chemical, toosendanin, isolated from *M. toosendan*, has been used to treat abdominal pain and as a digestive tract parasiticide in ancient China for about 1500 years [213].

Generally, these extracts or chemicals are comparatively safe to the environment, human beings, and natural enemies. It was reported that andiroba oil (including gedunin, 6α-acetoxygedunin, 7-deacetoxy-7-oxogedunin, 7-deacetylgedunin, 1,2-dihydro-3β-hydroxy-7-deacetoxy-7-oxogedunin, and andirobin) was not toxic in bioassays conducted with mice [214]. The ring-intact limonoid neoazedarachin B exhibited low toxicity in brine shrimp with an LC_50_ value of 0.0098 μM [215]. There were several reports about the safety of the extracts of *M. azedarach* to natural enemies. The aqueous leaf extracts of *M. azedarach* were reported to have no direct negative effects on the survival and foraging of parasitoids including *Cotesia plutellae* (Kurdjumov), *Diadromus collaris* (Gravenhorst), *Trichogramma evanescens* Westwood, *Aphidius ervi* Haliday, *Aphidius colemani* Viereck, *Eretmocerus eremicus* (Rose & Zolnerowich), and *Encarsia formosa* (Gahan) [138,139]. Another study revealed that unripe *M. azaderach* fruit extracts (1%, *w/w*, obtained by the CEPROCOR, Cordoba-Argentina) demonstrated no detrimental effects on *Eriopis connexa* (Germar) and could be compatible with *E. connexa* for pest control (Table 5) [216].

Normally, naturally derived plant extracts or chemicals are easily degraded and thus cause less residue to remain in the environment. For example, three days after the field application at five times the dose recommended by the manufacturer, the residue of salannin on strawberry (LOQ 0.01 mg/kg) was not detectable [217]. Another study demonstrated that toosendanin was easily degradable. At the recommended dose, the final residues of toosendanin detected by IC-ELISA were 0.009 mg/kg in cabbage and 0.043 mg/kg in tobacco. In soil, toosendanin residue was not detectable [218].

However, there were also some negative effects of these plant extracts or chemicals. Some extracts were reported to show a negative effect on rats. In detail, high doses of the crude water extract of *K. grandifoliola* reduced the Ca, P, Mg, and Zn levels of the bones and may have had an adverse effect on bone minerals in growing rats [219]. The methanol extracts of *K. ivorensis* were found to be relatively toxic, with an LD_50_ value of 549 mg/kg per body weight of the mice [220]. The ethanolic extract of *K. senegalensis* adversely affected the function of the liver and kidneys of rats [221,222,223]. *M. azedarach* was also reported to possess a potent pregnancy interceptive property on the rat [224]. Isolated chemicals, such as methyl angolensate and toosendanin, were also reported to possess some negative effects. For example, methyl angolensate could cause the inhibition of smooth muscle and reduce the propulsive action of the gastrointestinal tract in mice [225]. The traditional medicinal chemical toosendanin had serious hepatotoxicity [226]. Severe cytoplasmic vacuolation and nuclear shrinkage were found in the liver of toosendanin-treated zebrafish [227]. Further studies revealed that toosendanin was pregnancy-toxic to animals (Table 5) [228].

**Table 5:** Toxicity of isolated chemicals or plant extracts of the five genera (*Cipadessa*, *Entandrophragma*, *Guarea*, *Khaya*, and *Melia*) in Meliaceae on mice, aquatic organisms, and natural enemies.

Chemicals or Plant Extracts	Mice	Aquatic Organisms	Natural Enemies	Ref.
methyl angolensate	inhibition of smooth muscle, decrease of propulsive action of the gastrointestinal tract	-	-	[225]
toosendanin	serious hepatotoxicity, pregnancy-toxic	cytoplasmic vacuolation and nuclear shrinkage in liver of zebrafish	-	[226,227,228]
neoazedarachin B	-	low toxicity to brine shrimp, LC_50_ = 0.0059 μg/mL (48 h)	-	[215]
methanol extracts of *K. ivorensis*	relatively toxic, LD_50_ = 549 mg/kg	-	-	[220]
ethanolic extract of *K. senegalensis*	adverse effect on liver and kidney	-	-	[221,222,223]
chloroform fraction of *M. azedarach*	potent pregnancy interceptive property	-	-	[224]
andiroba oil (*C. guianensis* oil)	not toxic at 2000 mg/kg (14 d)	-	-	[214]
water extract of *K. grandifoliola*	adverse effect on bone minerals (at 500 mg/kg)	-	-	[219]
aqueous leaf extracts of *M. azedarach*	-	-	no direct negative effects on the survival and foraging of *Cotesia plutellae*, *Diadromus collaris*, *Trichogramma evanescens*, *Aphidius ervi*, *Aphidius colemani*, *Eretmocerus eremicus* and *Encarsia formosa*	[138,139]
*M. azaderach* unripe fruit extracts	-	-	no detrimental effects on *Eriopis connexa*	[216]

Briefly, several studies have been conducted on the environmental toxicity of the extracts of plants or isolated chemicals from the five genera (*Cipadessa*, *Entandrophragma*, *Guarea*, *Khaya*, and *Melia*) in Meliaceae. Further studies are needed to elucidate the environmental toxicity of some important insecticidal chemicals for their future application in the field.

## 6. Future Outlook

The unique insecticidal properties of insecticidal plants, particularly Meliaceae, which are safe both for the environment and natural enemies, and their compatibility with the agroecosystem emphasize their potential value in the integrated control of insect pests [195].

The use of toosendanin as an agricultural insecticide, with marked systemic properties, showing various activities including antifeeding, deterring, growth-inhibitory, contact poisoning, and stomach poisoning activities, was recorded about 2000 years ago in ancient China. There are still various commercial formulations of toosendanin on the market. In addition, there are other molecules with obvious insecticidal activity that deserve further attention. Like toosendanin, gedunin possesses various activities toward insects and exhibits good potential to be used as a lead compound for the development of novel insecticides. Other chemicals, such as khayasin and 12-deacetyltoosendanin, also deserve further attention. Their activities toward insects should be systemically evaluated, and their effects on nontarget organisms and the environment should also be further studied.

Recently, knowledge of the biosynthesis of important bioactive molecules in plants has become increasingly important. Understanding the biosynthetic pathways and their regulation has led to attempts to metabolically engineer bioactive molecules more successfully and more easily in economically important plants.

However, despite the intensive investigation of limonoids over several decades, the biosynthetic pathway of these triterpenoids is less understood. Enzymes involved in the biosynthesis of limonoids have been partially identified and characterized in some plant species. For example, AiOSC1 from the neem tree produces a single triterpene, tirucalla-7,24-dien-3β-ol, indicating the importance of its role in azadirachtin biosynthesis [229]. Toosendanin from *M. toosendan* was proposed to be synthesized in a manner similar to azadirachtin, by cyclizing the precursor 2,3-oxidosqualene into a tirucalla-7,24-dien-3β-ol as the scaffold, followed by scaffold rearrangements and the formation of the furan ring [5]. Lian et al. (2020) elucidated that MtOSC1 was a key enzyme in the production of triterpene tirucalla-7,24-dien-3β-ol, while MtOSC6 (a lupeol synthase) was a key enzyme in the production of lupeol in *M. toosendan*. The product of MtOSC1 was the precursor for the biosynthesis of toosendanin. This research provided a foundation for toosendanin biosynthesis and presented an important building block for the synthesis of insecticidal triterpenoids using the synthetic biology approach [230].

Using synthetic biology methods, the identified enzymes could be used to model a biosynthetic pathway to produce large quantities of insecticidal molecules, such as azadirachtin and toosendanin. Therefore, further research needs to be carried out for the clear elucidation of the biosynthetic pathway of certain highly effective plant-derived insecticidal molecules. The biosynthesis of important plant-derived insecticidal molecules in Meliaceae will be a significant research topic of interest in the coming years.

## Figures and Tables

**Figure 3:**
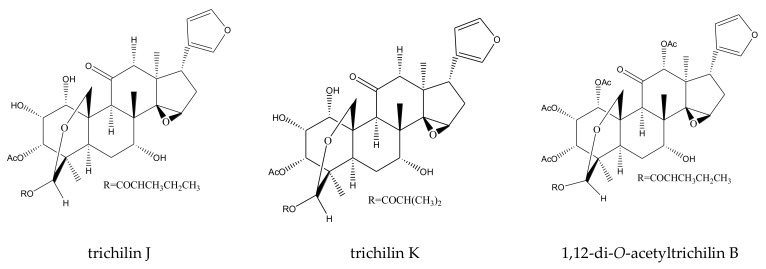

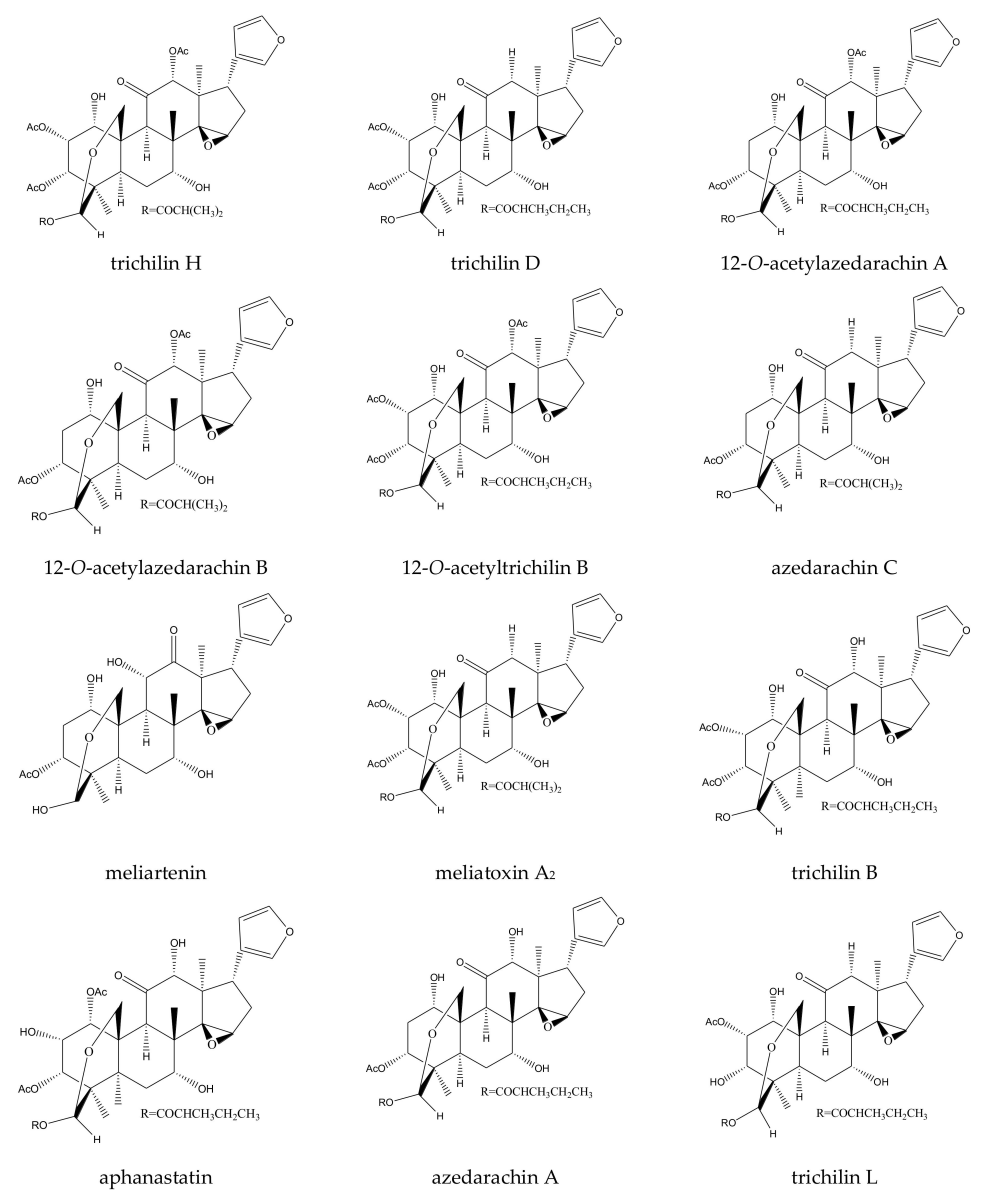

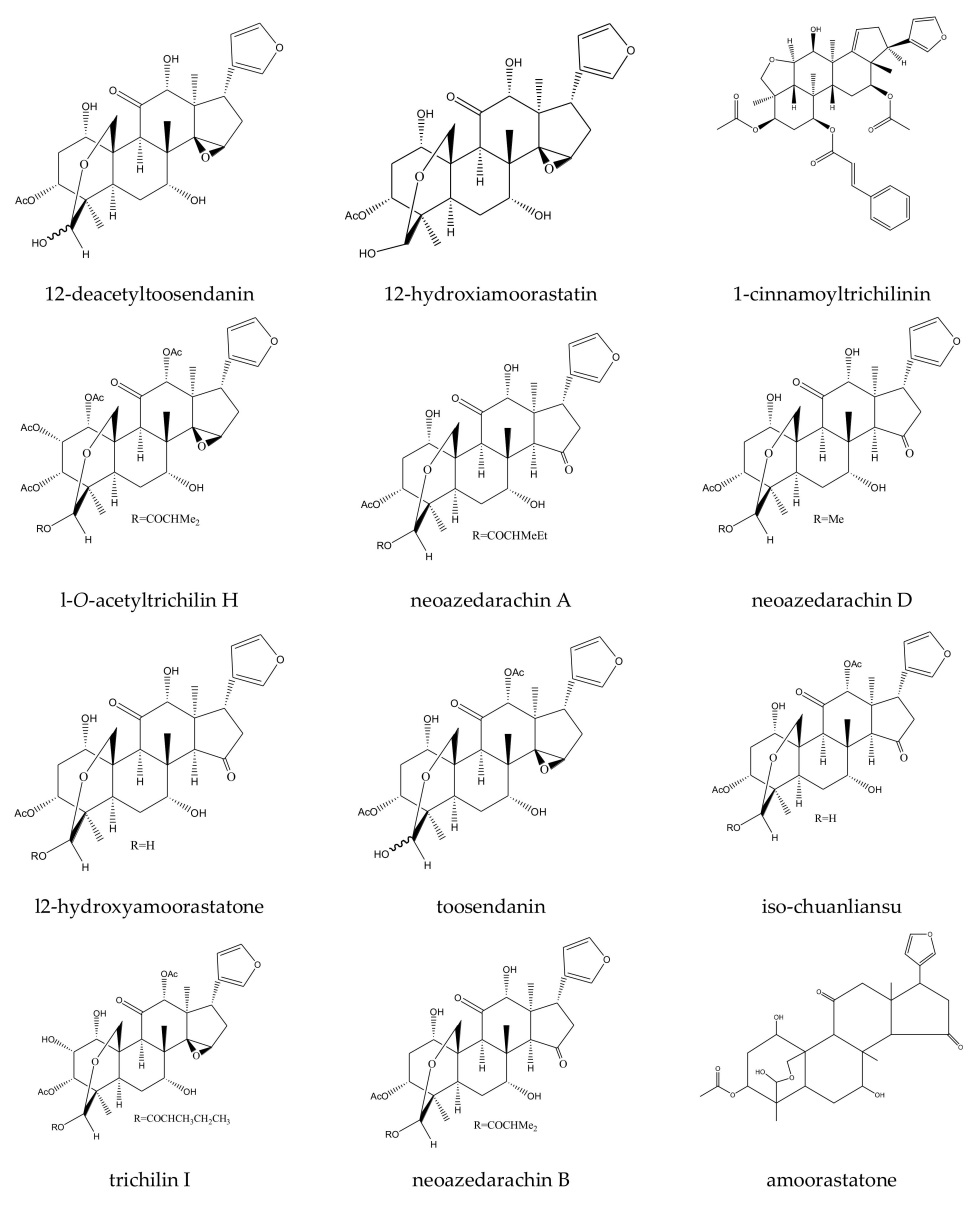

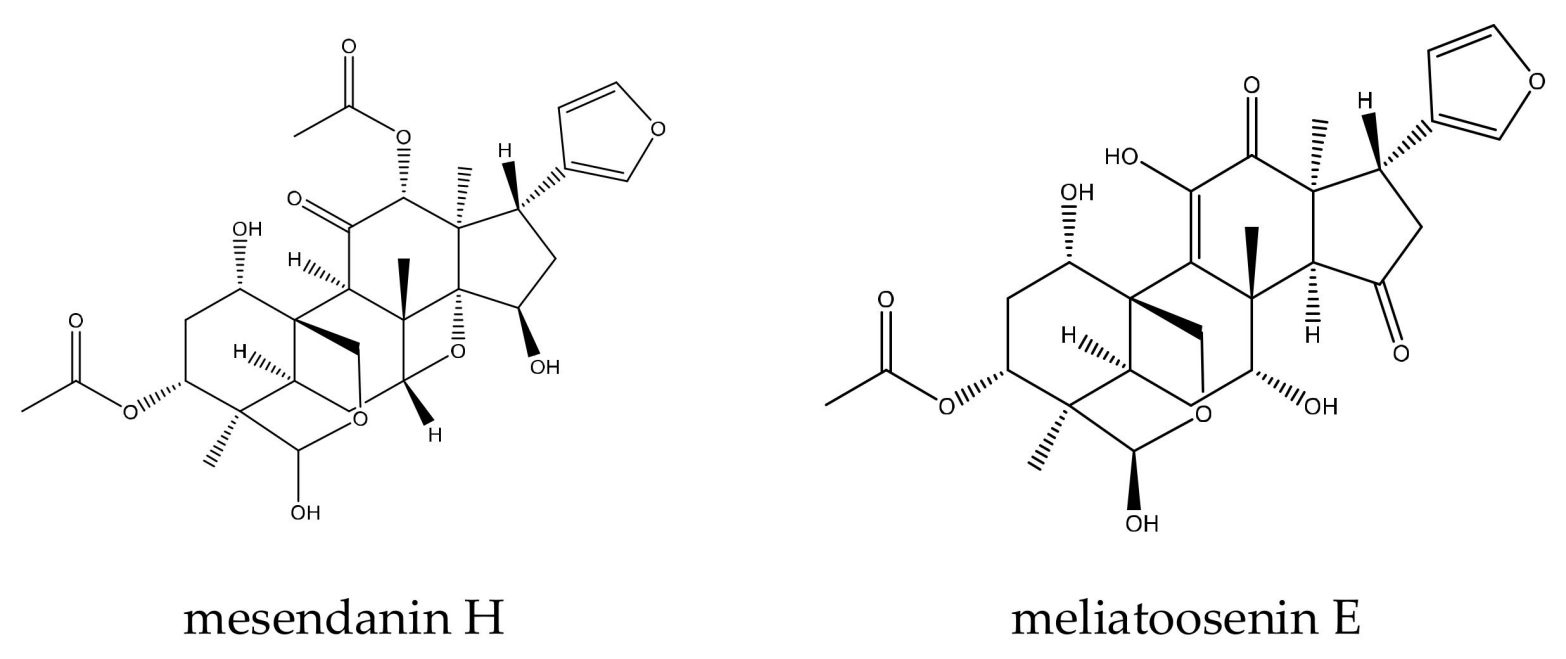
Structures of ring-intact limonoids: trichilin-class chemicals.

**Figure 4:**
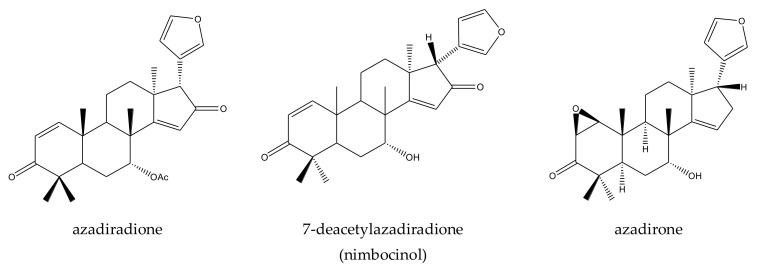
Structures of ring-intact limonoids: azadirone-class chemicals.

**Figure 5:**
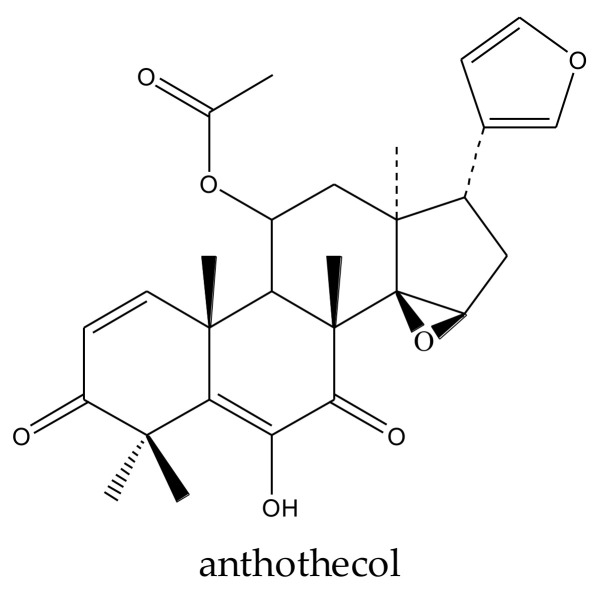
Structure of ring-intact limonoid: cedrelone-class chemical.

**Figure 6:**
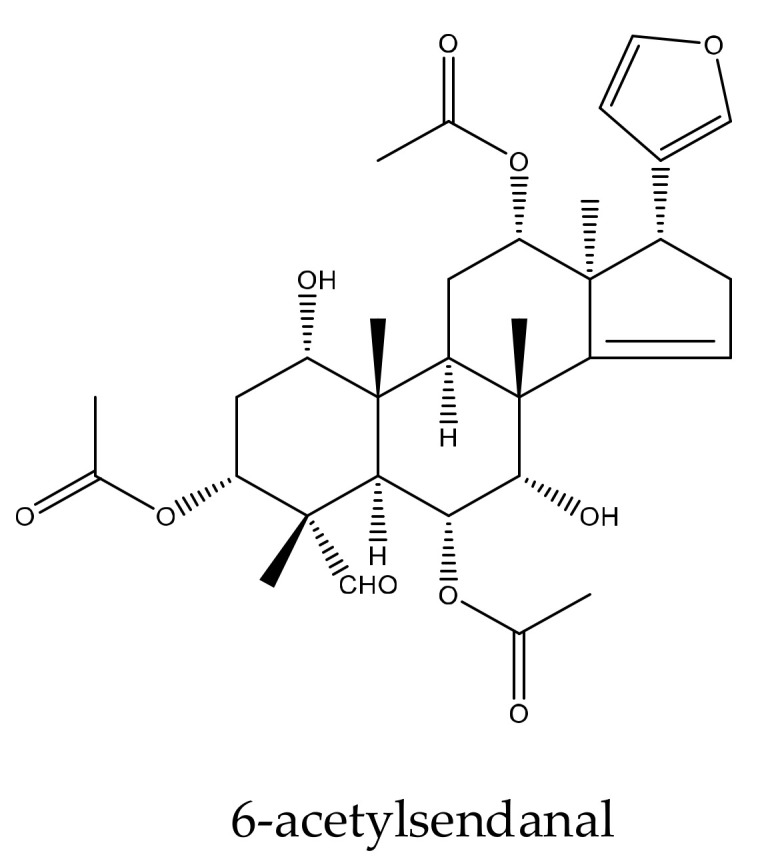
Structure of ring-intact limonoids: havanensin-class chemical.

**Figure 7:**
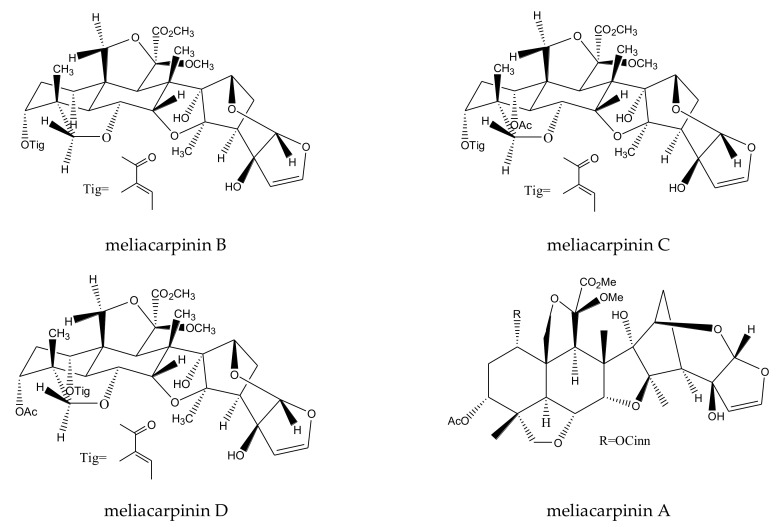
Structures of ring-seco limonoids: ring C-seco group (azadirachtinin/meliacarpinin-class chemicals).

**Figure 8:**
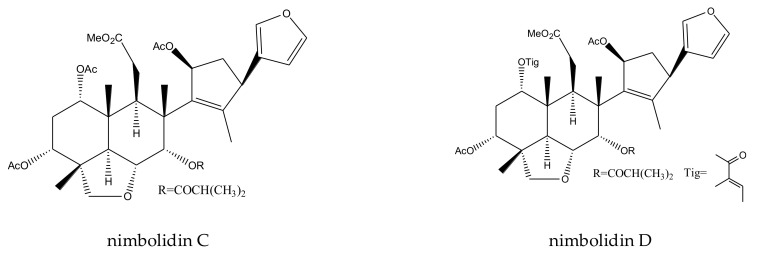

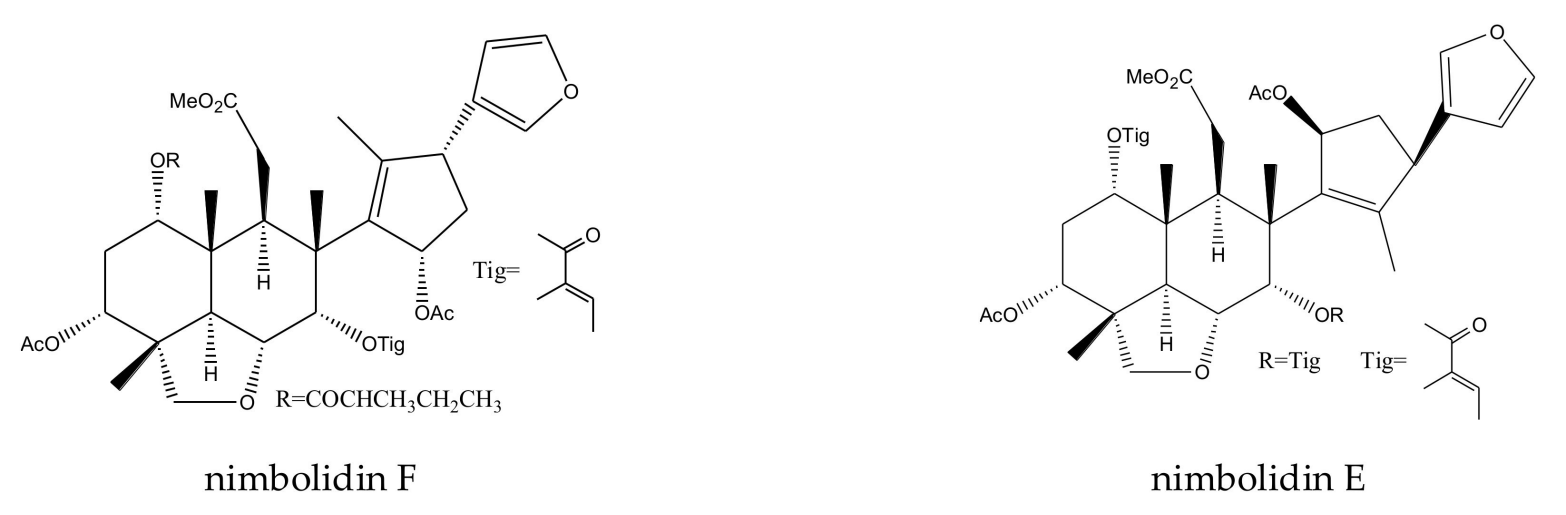
Structures of ring-seco limonoids: ring C-seco group (nimbolidin-class chemicals).

**Figure 9:**
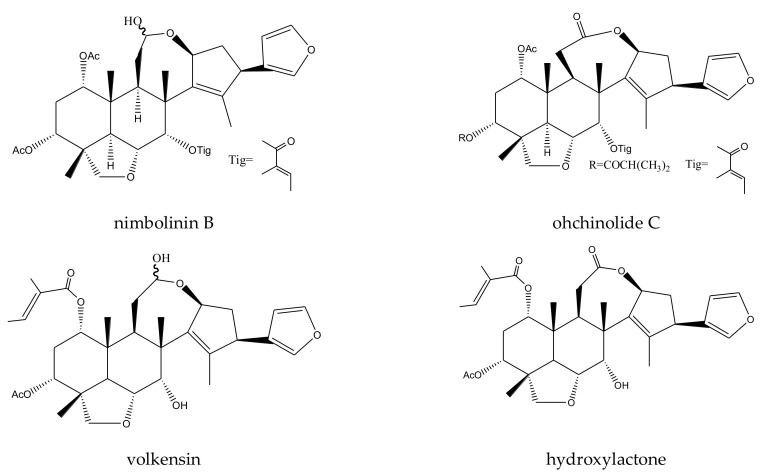
Structures of ring-seco limonoids: ring C-seco group (nimbolinin-class chemicals).

**Figure 10:**
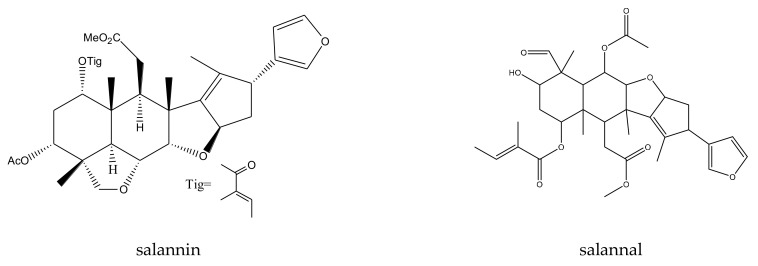

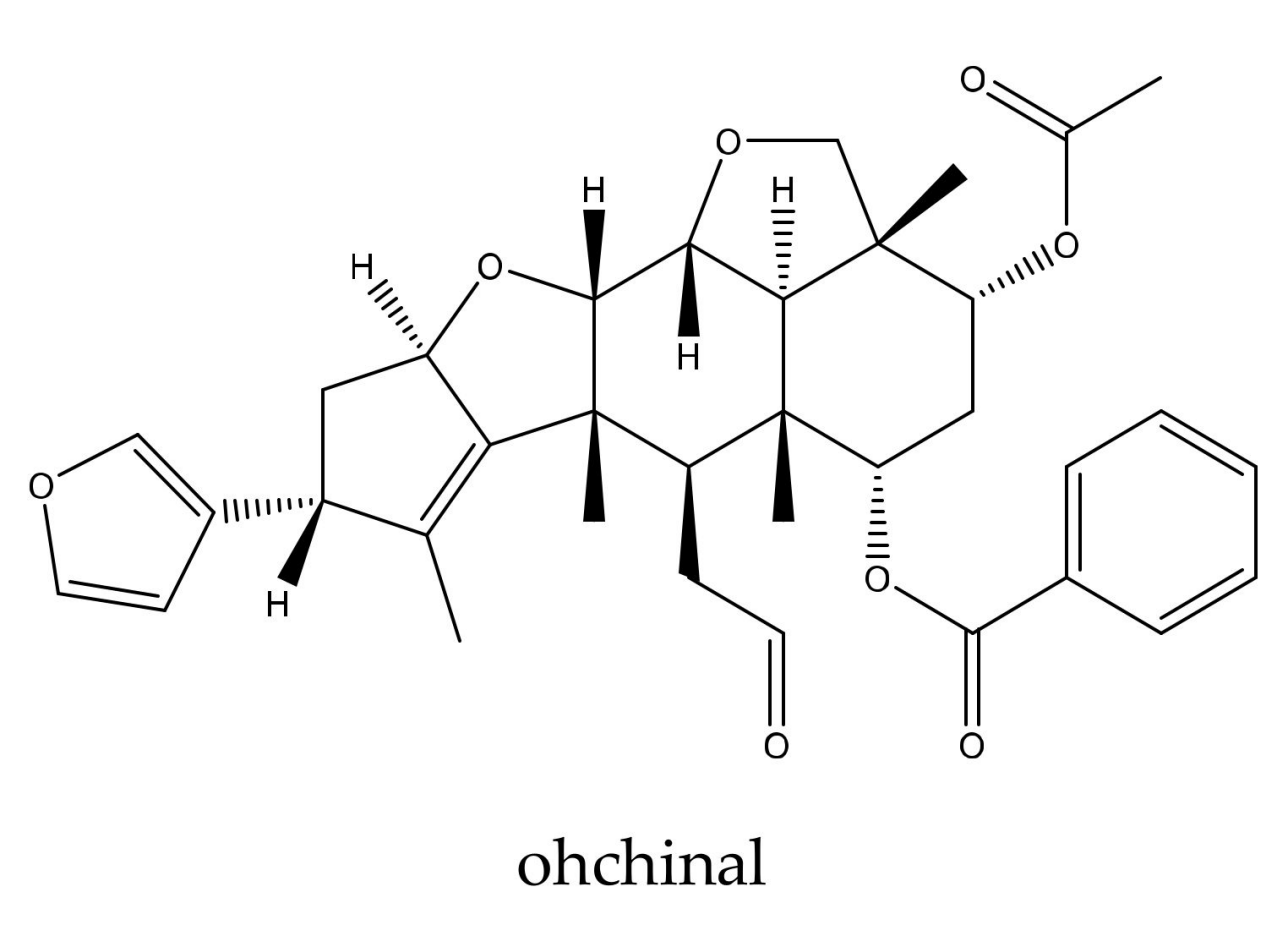
Structures of ring-seco limonoids: ring C-seco group (salannin-class chemicals).

**Figure 11:**
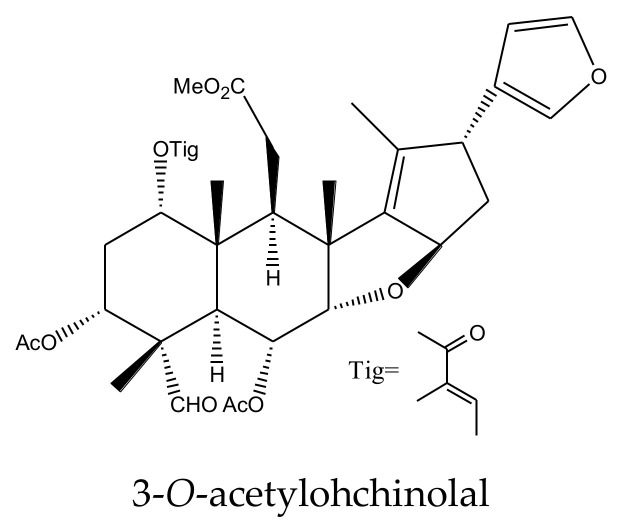
Structure of ring-seco limonoid: ring C-seco group (nimbin-class chemical).

**Figure 12:**
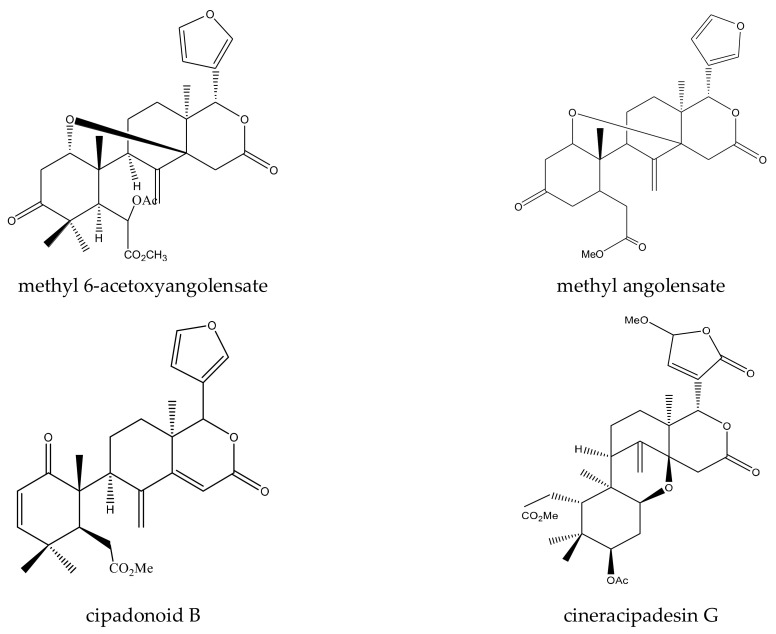
Structures of ring-seco limonoids: ring B,D-seco group (andirobin-class chemicals).

**Figure 13:**
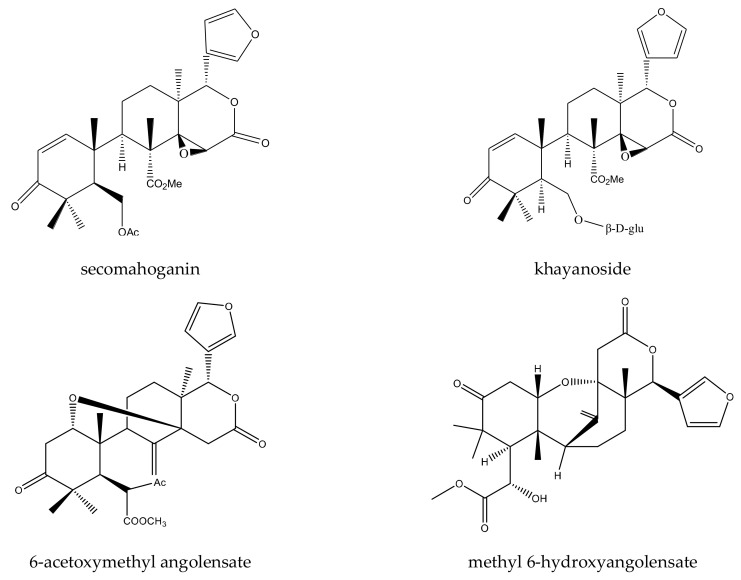
Structures of ring-seco limonoids: ring B,D-seco group (others).

**Figure 14:**
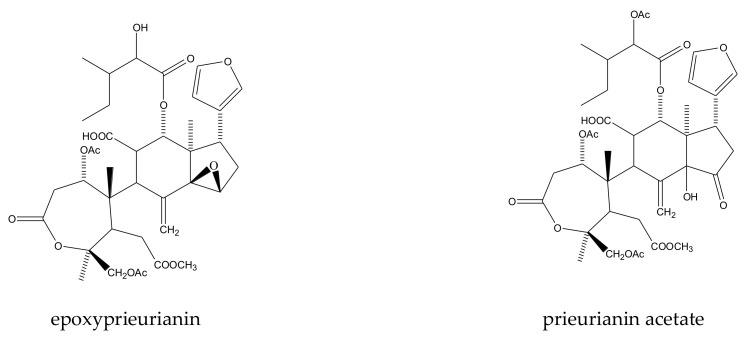

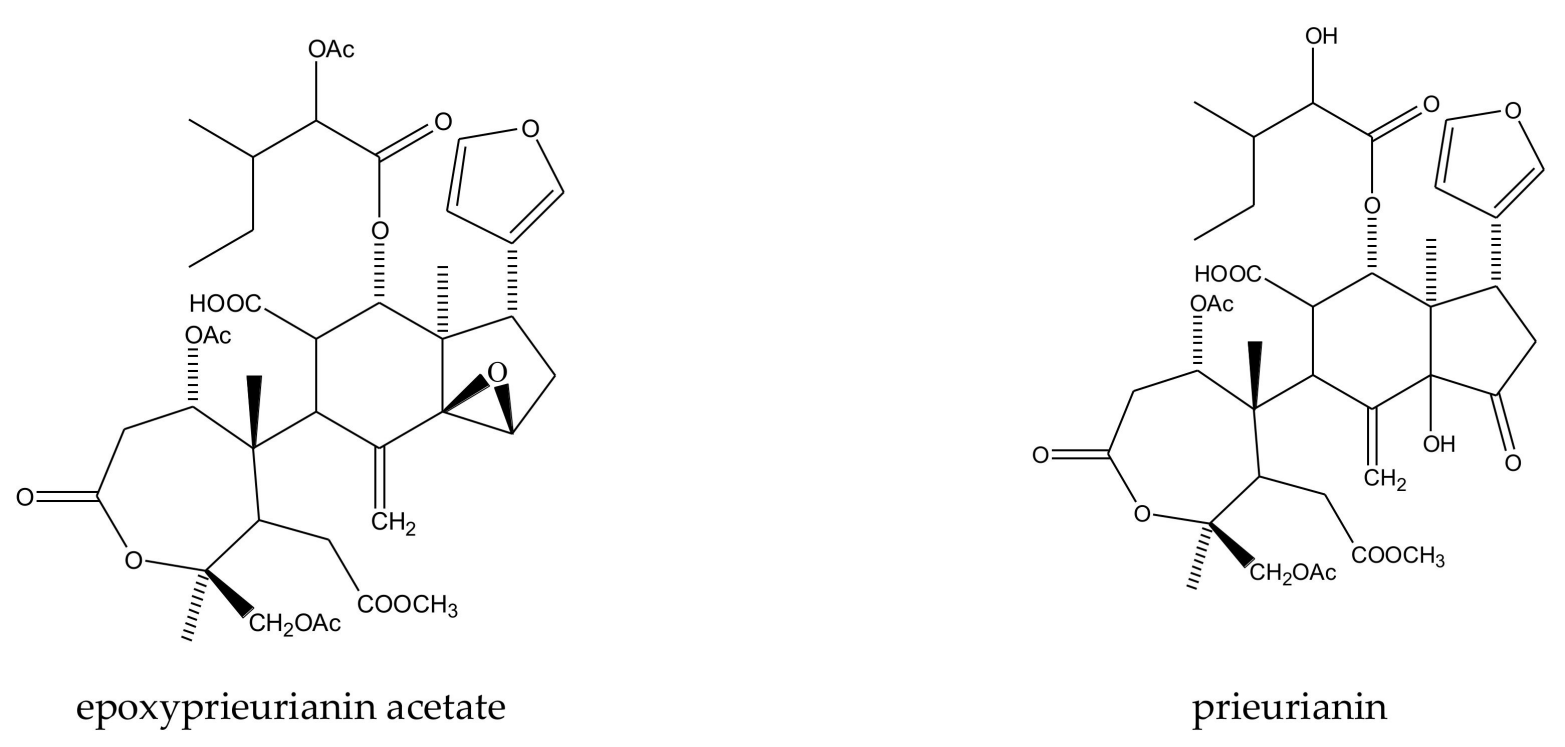
Structures of ring-seco limonoids: ring A,B-seco group (prieurianin-class chemicals).

**Figure 15:**
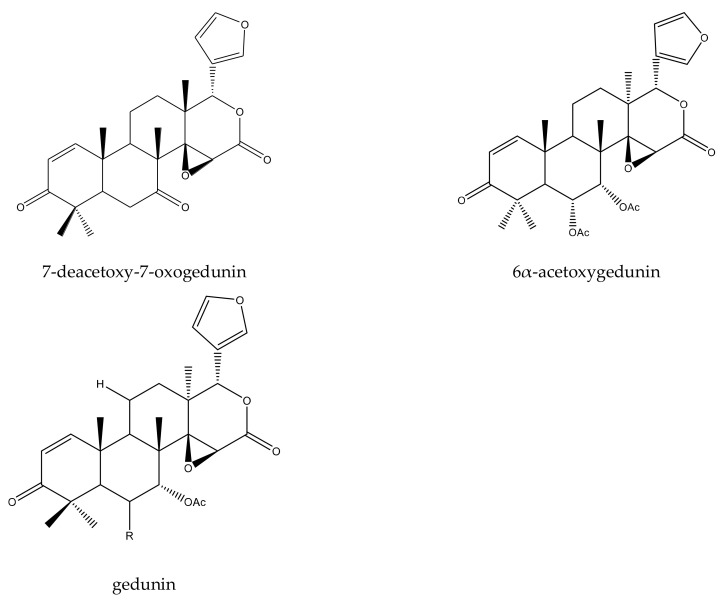
Structures of ring-seco limonoids: ring D-seco group chemicals.

**Figure 16:**
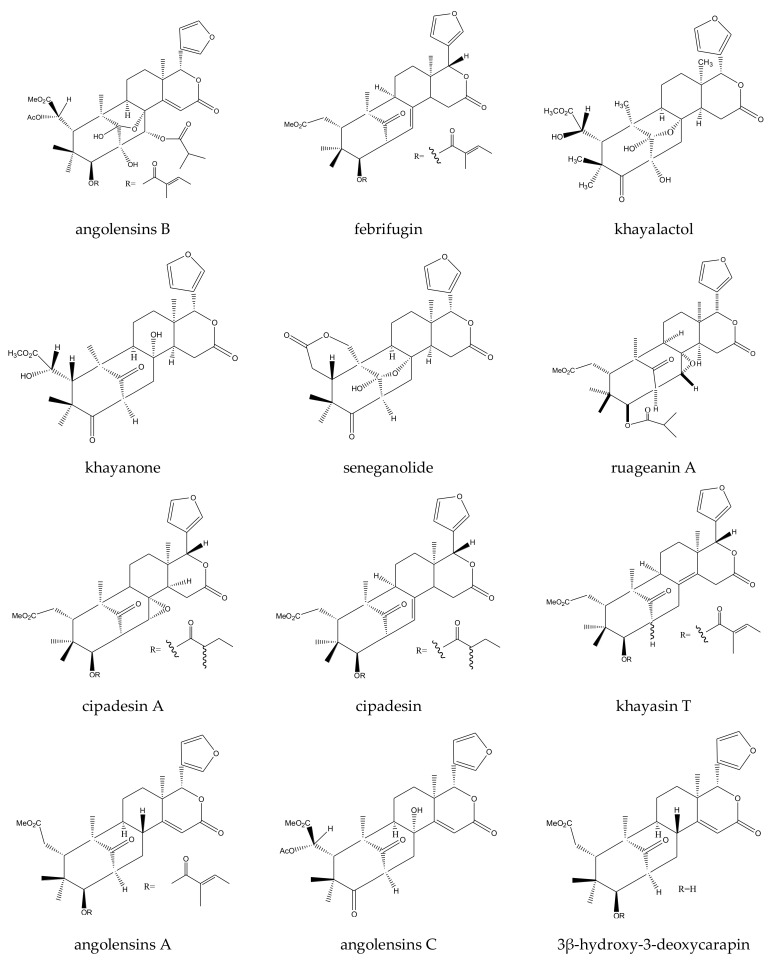

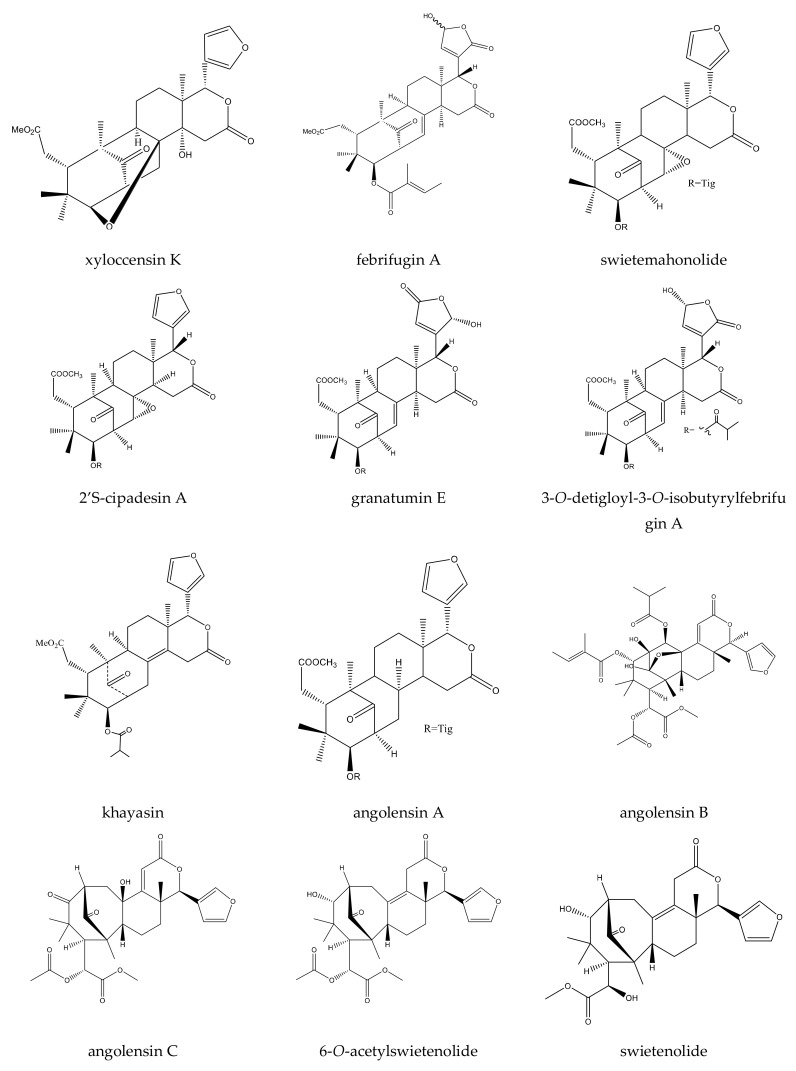
Structures of rearranged limonoids: ring 2,30-linkage group (mexicanolide-class chemicals).

**Figure 17:**
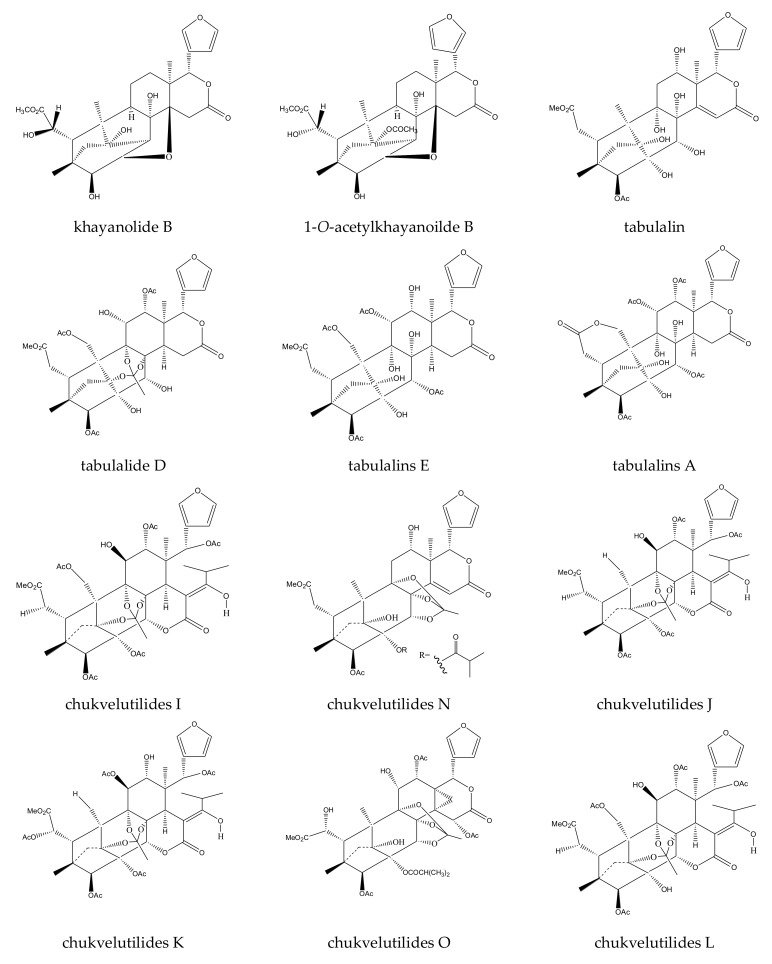

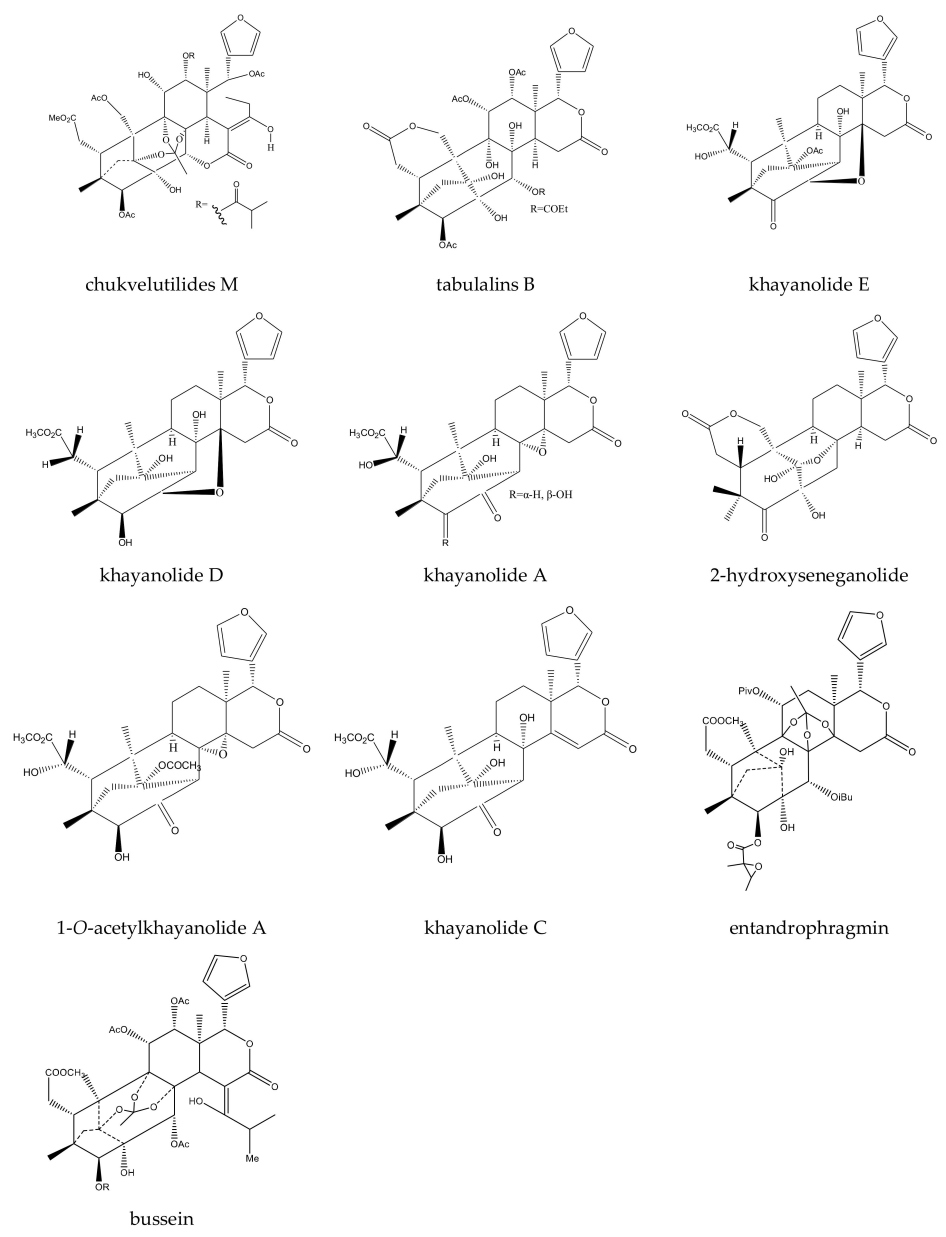
Structures of rearranged limonoids: ring 2,30-linkage group (phragmalin-class chemicals).

**Figure 18:**
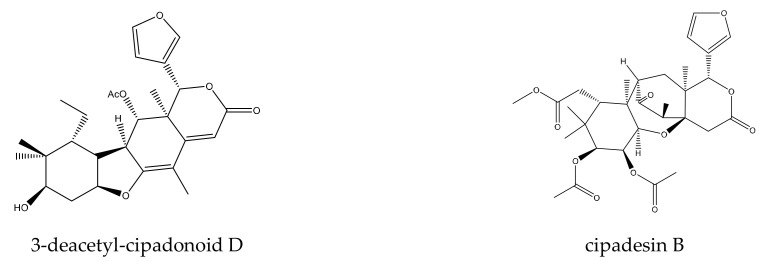
Structures of rearranged limonoids: ring 10,11-linkage group chemicals.

**Figure 19:**
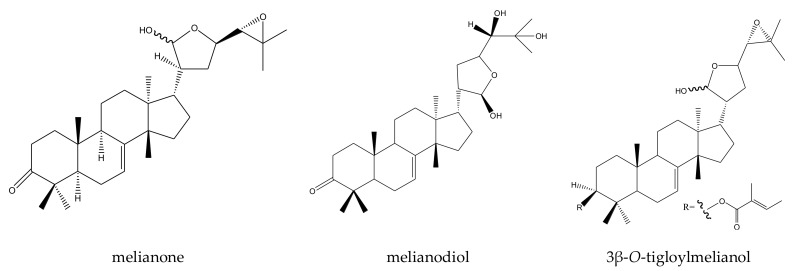
Structures of tetracyclic triterpenes: protolimonoids.

**Table 1:** The 22 insecticidal plant species of five genera in Meliaceae.

Family	Genus	Species
Meliaceae	*Cipadessa*	*Cipadessa baccifera* (Roth) Miq.
		*Cipadessa cinerascens* (Pell.) Hand-Mazz
	*Entandrophragma*	*Entandrophragma angolense* C. DC
		*Entandrophragma bussei* Harms ex Engl.
		*Entandrophragma caudatum* Sprague
		*Entandrophragma candolei* (Harms)
		*Entandrophragma delevoyi* (de Wild)
		*Entandrophragma cylindricum* (Sprague) Sprague
		*Entandrophragma spicatum* (C.DC.) Sprague
		*Entandrophragma macrophyllum* A. Chev.
	*Guarea*	*Guarea guidonia* (L.) Sleumer
		*Guarea grandiflora* ADC
		*Guarea thompsonii* Sprague et Hutch.
		*Guarea kunthiana* A. Juss
	*Khaya*	*Khaya anthotheca* (Welv.) C. DC
		*Khaya senegalensis* (Desr.) A. Juss.
		*Khaya grandifoliola* C. DC
		*Khaya ivorensis* A. Chev.
	*Melia*	*Melia azedarach* L.
		*Melia toosendan* Sieb. Et Zucc.
		*Melia dubia* Cav.
		*Melia volkensii* Gurke
